# The association of perioperative serum uric acid variation with in-hospital adverse outcomes in coronary artery bypass grafting patients

**DOI:** 10.3389/fcvm.2024.1364744

**Published:** 2024-10-01

**Authors:** Junyi Gao, Yi Cheng

**Affiliations:** ^1^ Department of Cardiovascular Medicine, Beijing Shijitan Hospital, Capital Medical University, Beijing, China; ^2^ Department of Diagnostic Ultrasound, Beijing Anzhen Hospital, Capital Medical University, Beijing, China

**Keywords:** serum uric acid, perioperative variation, coronary artery bypass grafting, in-hospital all-cause death, in-hospital fatal arrhythmia

## Abstract

**Background:**

Previous studies proposed the predictive value of baseline serum uric acid (SUA) in the prognosis of coronary artery bypass grafting (CABG) patients. The association of perioperative SUA variation with in-hospital adverse outcomes in CABG patients is unknown.

**Methods:**

A total of 2,453 patients were included in the study and were divided into four groups (G1–G4) according to perioperative SUA variation (ΔSUA) (G1, ΔSUA ≤ −90 μmol/L; G2, −90 μmol/L < ΔSUA < 0; G3, 0 ≤ ΔSUA < 30 μmol/L; G4, 30 μmol/L ≤ ΔSUA.) The basic characteristics and incidence of adverse outcomes were compared between the groups in the overall population and the subgroups. Multivariate logistic regression was performed to explore the association between perioperative SUA increases and adverse outcomes, and receiver operating characteristic analysis was used to obtain the cutoff value of SUA increases.

**Results:**

The patients had a mean age of 60.9 years and the majority were males (76.7%). In the group with the most significant increase in SUA (G4), incidences of in-hospital all-cause death and fatal arrhythmia were higher than in other groups in the overall population and the subgroups. Multivariate logistic regression showed that an increase in the SUA level of ≥30 µmol/L was significantly associated with in-hospital all-cause death and fatal arrhythmia, independent of the baseline SUA level and renal function. This association was significant in most subgroups for in-hospital fatal arrhythmia and in the ≥60 years, myocardial infarction, and female subgroups for in-hospital all-cause death. The cutoff values of SUA increases in the overall population were 54.5 µmol/L for in-hospital all-cause death and 42.6 µmol/L for in-hospital fatal arrhythmia.

**Conclusions:**

The perioperative SUA increase significantly correlated with a higher incidence of in-hospital all-cause death and fatal arrhythmia in CABG patients, independent of the baseline SUA level and renal function. Perioperative SUA variation may provide complementary information in the identification of patients potentially at risk.

## Introduction

Coronary artery bypass grafting (CABG) is one of the most effective revascularization strategies for severe coronary artery disease and can reduce mortality and improve the quality of life (1). Although the prognosis of CABG has been improved (2), the early identification of patients potentially at risk is crucial. Researchers have proposed the predictive value of several biological factors in the prognosis of CABG (3). As a final product of purine metabolism in humans, serum uric acid (SUA) is a traditional risk factor for cardiovascular disease (CVD) events (4).

Several studies have focused on the predictive value of elevated SUA in CABG prognosis. In these studies, the SUA level, either before or after surgery, was based on a single measurement. As far as we know, few studies have investigated the prognostic value of perioperative SUA. In 2023, Wu et al. found that elevated baseline SUA could predict the long-term prognosis in CABG patients (5). Memetoglu et al. proposed that elevated baseline SUA could predict atrial fibrillation (AF) after CABG (6). Lee et al. found that the preoperative SUA level can predict the occurrence of postoperative acute renal injury (AKI) after CABG (7). Hillis et al. and Lazzeroni et al. both proposed that the preoperative SUA level could independently predict long-term mortality after CABG (8, 9). In 2020, Shi et al. proposed that the preoperative SUA level was not associated with adverse outcomes. Instead, they found that it was associated with the in-hospital and 3-year adverse outcomes of CABG (10).

As shown in previous results, the predictive value of baseline SUA is controversial (11). First, baseline SUA is greatly influenced by several factors, such as race, gender, age, comorbidities, and gene polymorphisms (12, 13). Second, heterogeneity exists in the association between baseline SUA and CVD prognosis in different populations, such as older patients and diabetes mellitus (DM) patients (14–16). In addition, a significantly reduced baseline SUA has been reported to have an adverse effect on CVD prognosis, and the U-shaped curve made the prediction more difficult (17). The postoperative SUA level also has limitations as a predictor as it is influenced by the preoperative SUA level.

Recently, the academic community has begun to show interest in the prognostic value of SUA level variation. Cipolletta et al. found an increase in the incidence of cardiovascular events following a gout flare in a hyperuricemia population. Their result confirmed the association between an acute SUA increase and the following CVD events. In their opinion, the underlying mechanisms could be an inflammatory cascade or damage caused by reactive oxygen species (ROS) (18). Therefore, we speculated that an acute SUA increase after surgery may also have prognostic value. Our study aimed to investigate the association between an increase in perioperative SUA and in-hospital fatal arrhythmia and all-cause death in CABG patients.

## Materials and methods

### Patients

We retrospectively reviewed 3,507 patients undergoing CABG in the Department of Cardiac Surgery at Beijing Anzhen Hospital between 1 January 2017 and 31 December 2018. Patients who underwent concomitant surgery including valvular surgery (for severe tricuspid/mitral stenosis or regurgitation) and thoracic aortic surgery (for aortic dissection) (*n* = 262), patients who underwent emergency surgery for unstable hemodynamics such as an extremely poor cardiac function (*n* = 151), patients with a CABG history (*n* = 2), patients with missing data (*n* = 194), and patients with preoperative chronic kidney disease (CKD) and postoperative AKI (*n* = 445) were excluded.

**Figure 1:**
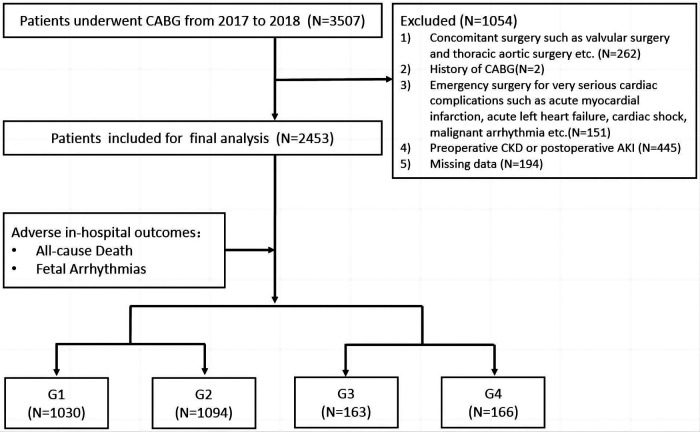
Flow diagram of patient selection and grouping. Group 1 (G1), ΔSUA ≤ −90 μmol/L; Group 2 (G2), −90 μmol/L < ΔSUA < 0; Group 3 (G3), 0 ≤ ΔSUA < 30 μmol/L; Group 4 (G4), 30 μmol/L ≤ ΔSUA. ΔSUA was defined as peak SUA within 48 h after surgery minus baseline SUA. CABG, coronary artery bypass graft; CKD, chronic kidney disease; AKI, acute kidney injury.

A total of 2,453 patients were finally included for further analysis. All patients were divided into four groups according to the median values of perioperative increases or decreases in SUA levels. ΔSUA was defined as peak SUA within 48 h after surgery minus baseline SUA. Group 1 (G1) and Group 2 (G2) included patients with SUA decreases of ≥90 µmol/L (ΔSUA ≤ −90) and <90 µmol/L (−90 < ΔSUA < 0), respectively. Group 3 (G3) and Group 4 (G4) included patients with SUA increases of <30 µmol/L (0 ≤ ΔSUA < 30) and ≥30  µmol/L (30 ≤ ΔSUA), respectively. Subgroup analyses were subsequently performed and patients were divided into subgroups according to age (<60 and ≥60 years), gender (male and female), body mass index (BMI) (<25 and ≥25 kg/m^2^), DM history (DM and non-DM), hypertension (HTN) history (HTN and non-HTN), and myocardial infarction (MI) history (MI and non-MI). A flow diagram of patient selection and grouping is shown in Figure 1.

Routine preoperative examinations were performed, including medical history collection, physical examinations, blood biochemical tests, electrocardiograms, and echocardiography. All patients received at least two blood biochemical tests within 48 h after surgery. Peak SUA level and creatinine (CREA) level within 48 h after the surgery were selected for calculation. The European System for Cardiac Operative Risk Evaluation (EuroSCORE) was calculated and a EuroSCORE ≥6 was recognized as high risk (19). High preoperative SUA (HUA) was defined as above 6 mg/dl (360 µmol/L) in women and above 7 mg/dl (420 µmol/L) in men (4). CKD was defined as having a history of renal failure or an admission estimated glomerular filtration rate (eGFR) of < 60 ml/min/1.73 m^2^ according to the Modification of Diet in Renal Disease (MDRD) formula (20). AKI was defined as a ≥0.3 mg/dl or ≥50% increase in CREA levels compared with baseline within the first 48 h after surgery (21).

We chose in-hospital all-cause death as the primary adverse outcome and it was defined as death from any cause during the period of hospitalization. In-hospital fatal arrhythmia was the secondary adverse outcome and included ventricular fibrillation, persistent ventricular tachycardia with hemodynamic disorders, and other arrhythmias with hemodynamic disorders (22, 23).

### Statistics

Continuous variables were described as mean ± standard deviation or median with interquartile range. Categorical variables were described as numbers and percentages. Univariate comparisons between groups were performed using the chi-squared test for categorical variables and an ANOVA for continuous variables, as appropriate. Multivariate logistic regression models were applied to explore the significant risk factors for in-hospital all-cause death and fatal arrhythmia in the overall population and subgroups. Potential covariates were included in the adjusted models according to univariate logistic regression analysis and clinical experience. Receiver operating characteristic (ROC) analysis was used to obtain the cutoff value of SUA increase in the overall population and subgroups. Analyses were performed using SPSS 26.0. (SPSS, Chicago, IL, USA); *p* < 0.05 was considered statistically significant.

The study was approved by the Ethics Committee of Beijing Anzhen Hospital, Capital Medical University. The protocol of this study was performed in accordance with the ethical standards of the 1964 Declaration of Helsinki and its later amendments. The committee waived the need for informed consent from the patients due to the retrospective nature of this study.

## Results

### Basic characteristics

The study included 2,453 patients with a mean age of 60.9 ± 8.6 years, and the majority were males (76.7%). Of these patients, 13.4% had increased perioperative SUA levels. In the overall patients, the four groups had comparable ages, a prevalence of HTN, stroke, AF, and an MI history. G4 patients had a higher prevalence of DM history, higher BMI, lower preoperative left ventricular ejection fraction (LVEF), and longer operation duration. The four groups had different preoperative and postoperative eGFRs (with or without significance) but were all within normal range. The proportions of high-risk patients evaluated by EuroSCORE were comparable between the four groups. The basic characteristics and incidences of adverse outcomes of the overall patients are summarized in Table 1.

**Table 1:** The basic characteristics and incidence of adverse outcomes in the overall population.

	All (*n* = 2,453)	G1 (*n* = 1,030)	G2 (*n* = 1,094)	G3 (*n* = 163)	G4 (*n* = 166)	*p*-value
General conditions						
Age (years)	60.9 ± 8.6	60.7 ± 8.8	61.3 ± 8.5	60.6 ± 8.3	59.5 ± 8.5^e^	0.045
Age ≥ 60 years (%)	54.5	53.1	56.9	54.0	48.8	0.142
Male (%)	76.7	79.8	73.5^a^	76.7	78.9	0.006
BMI (kg/m^2^)	25.5 ± 3.0	25.3 ± 3.0	25.4 ± 3.0	25.6 ± 3.1	26.4 ± 3.3^c,e,f^	<0.001
BMI ≥ 25 kg/m^2^ (%)	52.7	51.2	51.9	53.7	65.8^c,e,f^	0.006
Smoking history (%)	51.8	51.5	51.5	54.0	53.6	0.890
Drinking history (%)	28.7	31.1	25.8^a^	35.6^d^	26.5	0.009
HTN history (%)	65.1	67.3	63.3	66.3	63.3	0.246
DM history (%)	37.5	31.9	40.4^a^	43.6^b^	46.4^c^	<0.001
Insulin (%)	8.9	7.6	9.9	9.2	10.2	0.272
MI history (%)	28.2	27.6	28.2	25.8	33.7	0.363
PCI history (%)	12.8	14.1	11.6	12.3	12.7	0.400
Stroke/TIA history (%)	15.1	14.7	15.3	18.4	13.9	0.623
AF history (%)	2.4	2.4	2.3	3.1	2.4	0.950
COPD history (%)	0.4	0.3	0.3	0.6	1.2	0.446
EuroSCORE I ≥ 6 (%)	3.4	3.5	3.6	1.3	3.6	0.488
Preoperative and perioperative conditions						
TC (mmol/L)	4.15 ± 1.11	4.10 ± 1.11	4.18 ± 1.10	4.12 ± 1.05	4.29 ± 1.15^c^	0.147
TG (mmol/L)	1.38 (1.02–1.96)	1.39 (1.02–1.92)	1.35 (0.99–1.93)	1.42 (1.06–2.07)	1.51 (1.16–2.18)	0.050
HDL-C (mmol/L)	1.01 ± 0.24	1.00 ± 0.25	1.01 ± 0.24	0.99 ± 0.19	0.97 ± 0.22^e^	0.156
LDL-C (mmol/L)	2.52 ± 0.94	2.47 ± 0.93	2.55 ± 0.96	2.44 ± 0.80	2.68 ± 0.94^c,f^	0.018
Creatinine (μmol/L)	73.0 ± 14.0	75.5 ± 14.0	71.2 ± 13.9^a^	70.9 ± 13.0^b^	71.7 ± 13.0^c^	<0.001
eGFR (ml/min/1.73 m^2^)	92.0 ± 12.5	90.5 ± 13.0	92.7 ± 12.2^a^	93.5 ± 11.8^b^	94.5 ± 11.4^c^	<0.001
eGFR ≥ 90 ml/min/1.73 m^2^ (%)	62.5	57.3	65.0^a^	69.9^b^	70.5^c^	<0.001
SUA (μmol/L)	324.9 ± 83.2	364.0 ± 77.1	301.4 ± 73.7^a^	278.7 ± 80.4^b,d^	282.3 ± 79.8^c,e^	<0.001
HUA (%)	14.6	24.6	7.7^a^	6.1^b^	6.0^c^	<0.001
Na^+^ (mmol/L)	140.375 ± 2.461	140.392 ± 2.249	140.330 ± 2.622	140.621 ± 2.521	140.320 ± 2.566	0.546
Cl^−^ (mmol/L)	102.528 ± 2.897	102.555 ± 2.754	102.493 ± 2.976	102.564 ± 2.847	102.549 ± 3.287	0.962
K^+^ (mmol/L)	4.124 ± 0.355	4.113 ± 0.346	4.142 ± 0.359	4.066 ± 0.363^d^	4.134 ± 0.370	0.040
Mg^2+^ (mmol/L)	0.903 ± 0.079	0.904 ± 0.078	0.904 ± 0.079	0.897 ± 0.076	0.897 ± 0.084	0.472
Ca^2+^ (mmol/L)	2.345 ± 0.109	2.349 ± 0.112	2.343 ± 0.108	2.345 ± 0.098	2.341 ± 0.108	0.570
hsCRP(mg/L)	1.53 (0.62–4.09)	1.46 (0.59–3.86)	1.59 (0.61–4.12)	1.48 (0.76–5.27)	1.63 (0.74–4.36)	0.104
LVEF (%)	60.2 ± 9.3	60.7 ± 8.9	60.3 ± 9.2	60.8 ± 8.6	56.5 ± 11.3^c,e,f^	<0.001
LVEF ≥ 50% (%)	87.5	88.4	88.8	88.3	72.9^c,e,f^	<0.001
Preoperative heart rate (bpm)	75.4 ± 10.2	75.2 ± 1.5	75.6 ± 10.1	75.6 ± 9.8	75.1 ± 9.8	0.800
Preoperative PR (ms)	163.3 ± 26.0	163.1 ± 26.1	162.9 ± 25.5	164.6 ± 27.2	165.5 ± 27.8	0.607
Preoperative QTc (ms)	433.8 ± 34.3	434.5 ± 34.8	433.2 ± 34.1	434.1 ± 33.1	433.6 ± 34.6	0.844
Preoperative SBP (mmHg)	128.8 ± 16.1	127.3 ± 15.4	128.8 ± 16.4^a^	132.2 ± 16.1^b,d^	133.8 ± 16.9^c,e^	<0.001
Preoperative DBP (mmHg)	75.0 ± 9.8	74.5 ± 9.9	75.4 ± 9.7^a^	74.8 ± 9.4	74.9 ± 9.8	0.223
Blood loss (ml)	700 (600–1,000)	700 (600–950)	700 (590–900)	800 (600–1,000)	800 (600–1,000)	0.120
Postoperative conditions						
Duration of surgery (h)	4.25 ± 0.90	4.20 ± 0.83	4.24 ± 0.94	4.51 ± 0.95^b^	4.45 ± 0.96^c^	0.010
CREA (μmol/L)	76.3 ± 16.9	74.4 ± 17.5	76.6 ± 16.6^a^	79.4 ± 15.4^b,d^	83.8 ± 13.7^c,e,f^	<0.001
eGFR (ml/min/1.73 m^2^)	88.2 ± 14.8	90.2 ± 14.8	87.5 ± 14.7^a^	85.9 ± 13.9^b^	82.7 ± 14.3^c,e,f^	<0.001
SUA (μmol/L)	249.7 ± 80.2	222.9 ± 65.7	251.9 ± 73.9^a^	291.6 ± 80.3^b,d^	360.2 ± 87.5^c,e,f^	<0.001
Na^+^ (mmol/L)	139.918 ± 3.983	139.584 ± 3.771	139.996 ± 3.950^a^	140.509 ± 4.701^b^	140.877 ± 4.464^c,e^	<0.001
Cl^−^ (mmol/L)	104.575 ± 3.526	104.446 ± 3.521	104.656 ± 3.379	104.666 ± 3.947	104.750 ± 4.031	0.487
K^+^ (mmol/L)	4.143 ± 0.375	4.148 ± 0.366	4.139 ± 0.379	4.164 ± 0.397	4.116 ± 0.376	0.639
Mg^2^ ^+^ (mmol/L)	0.834 ± 0.131	0.835 ± 0.133	0.838 ± 0.131	0.814 ± 0.121^d^	0.827 ± 0.127	0.171
Ca^2^ ^+^ (mmol/L)	1.992 ± 0.149	1.992 ± 0.150	1.993 ± 0.149	1.994 ± 0.141	1.988 ± 0.156	0.986
In-hospital adverse outcomes						
Fatal arrhythmia (%)	1.6	1.3	1.6	0.6	5.4^c,e,f^	0.009
All-cause death (%)	1.0	1.0	0.8	0.6	2.4	0.385

BMI, body mass index; HTN, hypertension; DM, diabetes mellitus; MI, myocardial infarction; PCI, percutaneous coronary intervention; TIA, transient ischemic attack; AF, atrial fibrillation; COPD, chronic obstructive pulmonary disease; EuroSCORE, European System for Cardiac Operative Risk Evaluation; TG, triglyceride; HDL-C, high-density lipoprotein cholesterol; LDL-C, low-density lipoprotein cholesterol; CREA, creatinine; eGFR, estimated glomerular filtration rate; SUA, serum uric acid; HUA, high preoperative serum uric acid; hsCRP, high-sensitivity C-reactive protein; LVEF, left ventricular ejection fraction; PR, P-R interval; QTc, Q-T corrected interval; SBP, systolic blood pressure; DBP, diastolic blood pressure.

^a^ Significant difference between G1 and G2.

^b^ Significant difference between G1 and G3.

^c^ Significant difference between G1 and G4.

^d^ Significant difference between G2 and G3.

^e^ Significant difference between G2 and G4.

^f^ Significant difference between G3 and G4.

**Figure 2:**
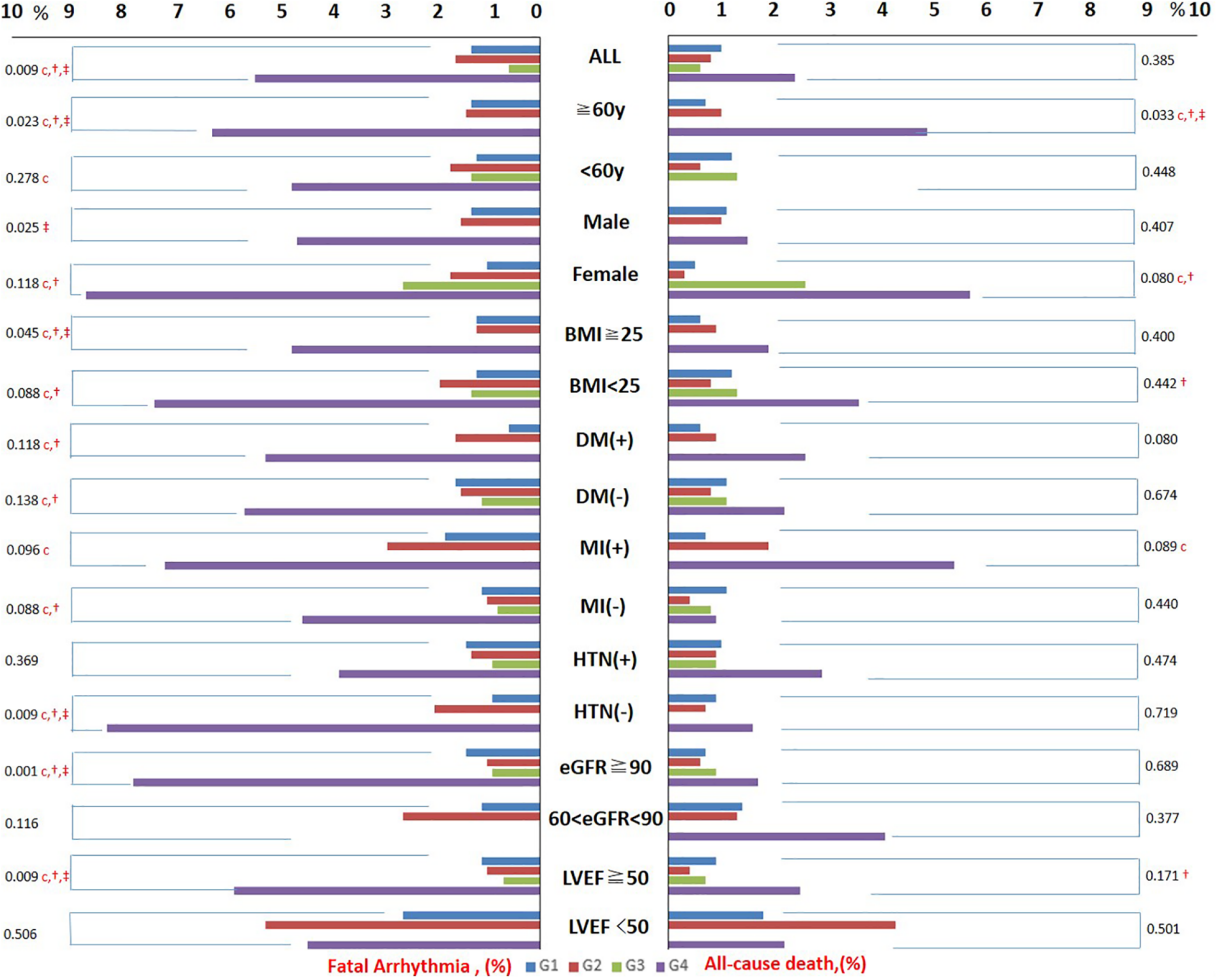
The incidence of adverse outcomes from G1 to G4 in the overall population and subgroups. Trends of in-hospital all-cause death and in-hospital fatal arrhythmia from G1 to G4 in all subgroups were similar. G4 had the highest incidence of in-hospital all-cause death and in-hospital fatal arrhythmia in most subgroups with or without significance. BMI, body mass index; HTN, hypertension; DM, diabetes mellitus; MI, myocardial infarction. The character “c” represents a significant difference between G1 and G4, “†” represents a significant difference between G2 and G4, and “‡” represents a significant difference between G3 and G4.

Incidences of in-hospital all-cause death from G1 to G4 were 1.0%, 0.8%, 0.6%, and 2.4%, respectively (*p* = 0.385). Incidences of in-hospital fatal arrhythmia from G1 to G4 were 1.3%, 1.6%, 0.6%, and 5.4%, respectively (*p* = 0.009). Trends of in-hospital all-cause death and in-hospital fatal arrhythmia from G1 to G4 in the subgroups were similar: G4 had the highest incidence of in-hospital all-cause death and in-hospital fatal arrhythmia in most subgroups, with or without significance. Incidences of adverse outcomes from G1 to G4 in the overall patients and subgroups are shown in Table 1, Supplementary Tables 1–12, and Figure 2.

### Results from the multivariate logistic regression analysis

#### In-hospital all-cause death

**Figure 3:**
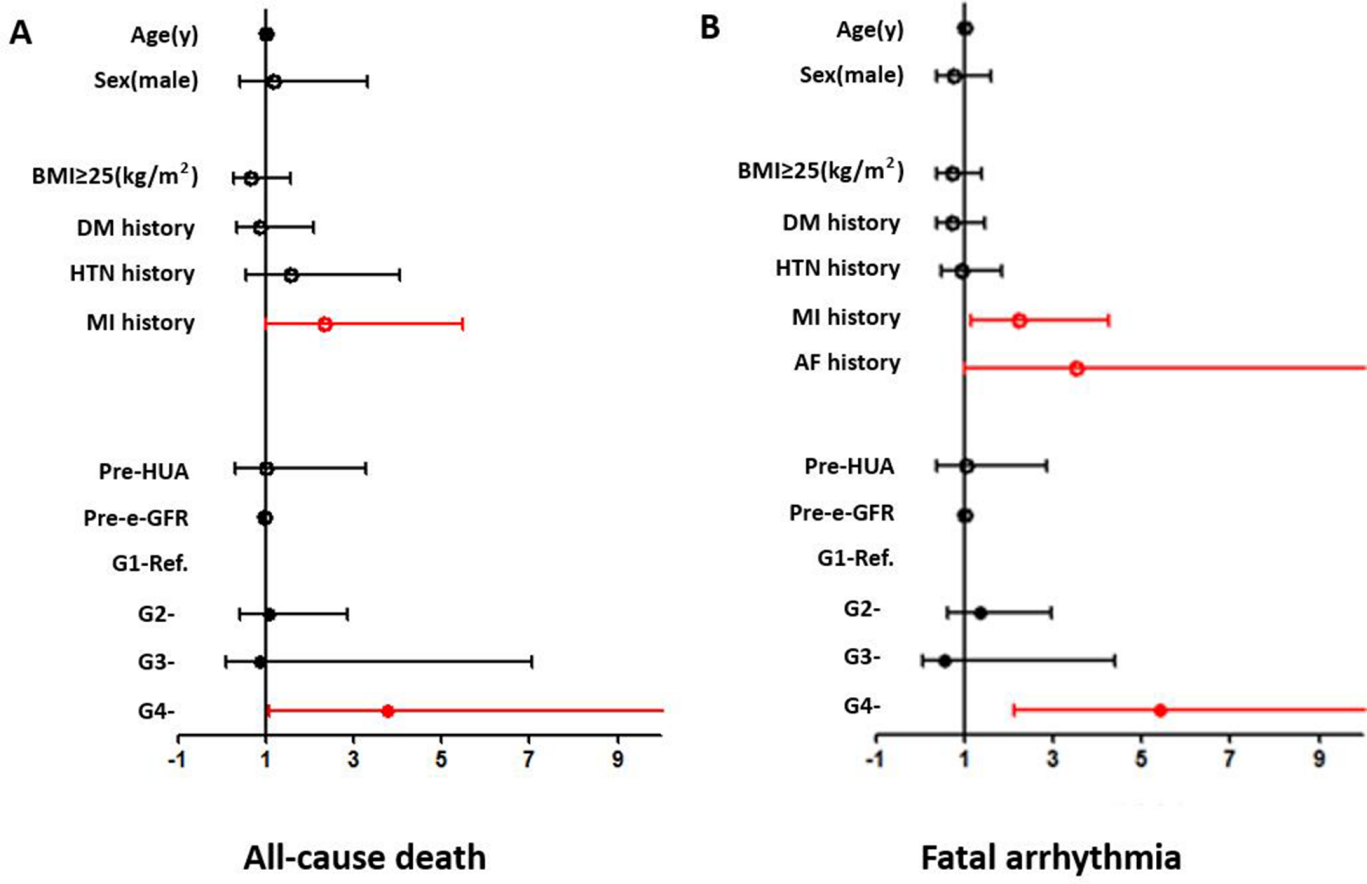
Forest plots from the multivariate logistic regression analysis of risk factors associated with the incidence of all-cause death (**A**) and fatal arrhythmia (**B**) in the overall population. An SUA level increase of ≥30 µmol/L (OR = 3.771, *p* = 0.039) and MI history (OR = 2.352, *p* = 0.048) were independent risk factors for all-cause death. An SUA level increase of ≥30 µmol/L (OR = 5.416, *p* < 0.001) and MI history (OR = 2.219, *p* = 0.016) were independent risk factors for fatal arrhythmia.

Age, gender, BMI ≥25 kg/m^2^, preoperative eGFR, HUA, HTN history, DM history, MI history, and perioperative SUA variation strata (G1–G4) were included as covariates. An SUA level increase of ≥30 µmol/L [G4, odds ratio (OR) = 3.771 (1.070–13.285) (G1 as reference), *p* = 0.039] and MI history [G4, OR = 2.352 (1.008–5.488), *p* = 0.048] were independent risk factors in the overall patients (shown in Figure 3).

An SUA increase of ≥30 µmol/L was a significant risk factor in several subgroups, including the ≥60 years (OR = 16.063, *p* = 0.001), female (OR = 22.436, *p* = 0.030), and MI (OR = 18.429, *p* = 0.006) subgroups. MI history was a significant risk factor for in-hospital all-cause death in the ≥60 years (OR = 5.077, *p* = 0.005), male (OR = 3.024, *p* = 0.025), and BMI ≥25 kg/m^2^ (OR = 4.291, *p* = 0.029) subgroups. Age was also a significant risk factor in the MI subgroup (OR = 1.087, *p* = 0.044) (shown in Supplementary Figure 1).

#### In-hospital fatal arrhythmia

Age, gender, BMI ≥25 kg/m^2^, preoperative eGFR, HUA, HTN history, DM history, MI history, AF history, and perioperative SUA variation strata (G1–G4) were included as covariates. Multivariate logistic regression analysis showed that MI history [OR = 2.219 (1.157–4.257), *p* = 0.016] and an SUA increase of ≥30 µmol/L [G4, OR = 5.416 (2.132–13.755) (G1 as reference), *p* < 0.001] were independent risk factors in the overall patients (shown in Figure 3).

An SUA increase of ≥30 µmol/L was a significant risk factor in most subgroups (shown in Supplementary Figure 2). MI was a significant risk factor in the female, non-DM, and HTN subgroups. AF was a significant risk factor in BMI <25 kg/m^2^, non-HTN, and non-MI subgroups. Age was a significant risk factor in the non-HTN subgroup and gender was also a significant risk factor in the MI subgroup (shown in Supplementary Figure 2).

### ROC analysis

**Figure 4:**
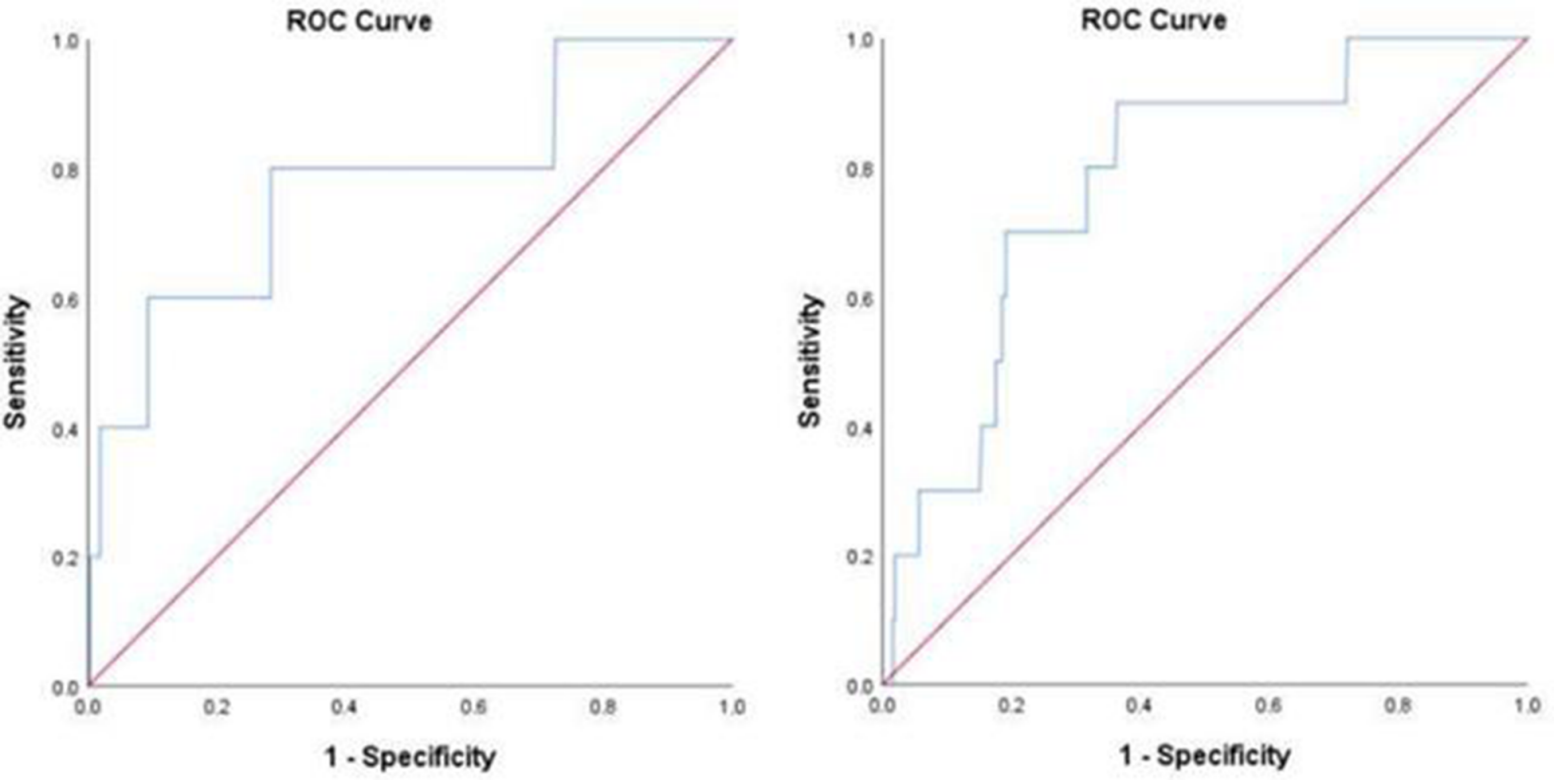
ROC analysis for the prediction of all-cause death (**A**) and fatal arrhythmia (**B**) by perioperative uric acid increase in the overall population. (**A**) AUC 0.776, 95% confidence interval (CI) (0.539–1.000) (*p* = 0.034); cutoff value, 54.5 μmol/L. (**B**) AUC 0.781, 95% CI (0.654–0.908) (*p* = 0.002); cutoff value, 42.6 μmol/L.

As for in-hospital all-cause death, the cutoff value was 54.5 µmol/L and the area under the curve (AUC) was 0.776 (0.539–1.000, *p* = 0.034) in the overall patients. The cutoff values were 53.4, 99.4, and 52.5 µmol/L in the age ≥60 years, DM, and MI subgroups, respectively (shown in Figure 4 and Supplementary Figure 3).

Regarding in-hospital fatal arrhythmia, the cutoff value was 42.6 µmol/L and the AUC was 0.781 (0.654–0.908, *p* = 0.002) in the overall patients. The cutoff values in the subgroups varied from 42.6 to 75.6 µmol/L (shown in Figure 4 and Supplementary Figure 4).

## Discussion

In the overall patients, the in-hospital all-cause death rate was 1.0% and the in-hospital fatal arrhythmia rate was 1.6%, which were consistent with a previous study (2). The most important result was that the direction of SUA change (not the absolute value of change) was associated with adverse outcomes. Incidences of in-hospital all-cause death and fatal arrhythmia in G4 were higher than in the other groups in the overall patients, and this trend was consistent in most subgroups. Multivariate logistic regression analysis in the overall population showed that a perioperative SUA level increase of ≥30 µmol/L was significantly associated with in-hospital all-cause death (OR = 3.771) and in-hospital fatal arrhythmia (OR = 5.416). This association was independent of the preoperative SUA level and renal function.

Previous studies proposed that the effect of SUA on CVD outcomes could be influenced by several factors, such as age (24), gender (15), BMI (25), and DM history (14). Therefore, we divided the patients into subgroups based on age, gender, BMI, DM history, HTN history, and MI history for further analysis. Results confirmed that there was a discrepancy in the correlation between a perioperative SUA increase and adverse outcomes between the subgroups. The association of an SUA increase with all-cause death was more prominent in the older, female, and MI subgroups. As for in-hospital fatal arrhythmia, an SUA increase showed a significant association with adverse outcomes in almost all the subgroups except for the HTN history subgroup. HTN might conceal the effect of SUA as they have similar mechanisms in CVD events.

An important question that needs to be answered is the source of the SUA increase. Increased production (endogenous or exogenous) and decreased excretion are the main reasons for an SUA increase (26). UA is a metabolite from purines, and the main exogenous source of purines is food intake, including fatty meat, seafood, and fructose, and the main endogenous sources are nucleic acids released from dying cells (4). UA is finally excreted by the kidneys (65%–75%) and intestines (25%–35%). Renal failure and the use of certain drugs increase the SUA level by decreasing renal excretion (26).

In our study, we selected the maximum SUA level within 48 h after surgery to minimize the effect of food intake. We assume that the main reason for the increase in SUA might be tissue damage and massive cell death (27). In the study by Cipolletta et al., subsequent adverse CVD outcomes were associated with an earlier gout flare (18). Their study indicated the effect of an acute SUA increase, which supported our speculation.

Although we excluded patients with preoperative CKD and postoperative AKI, the effect of a perioperative change in renal function should not be ignored. Patients in G1, G2, and G3 had comparable pre- and post-surgery renal function (shown in Table 1). Although the decrease in renal function in G4 was the most obvious among the four groups (from 94.5 ± 11.4 to 82.7 ± 14.3 ml/min/1.73 m^2^), pre- and post-surgery eGFRs in this group were all in the normal range. In addition, we assume that a decrease of 10 ml/min/1.73 m^2^ in renal function is of no clinical significance. As for the electrolytes, the absolute values before and after surgery in the four groups were similar (with or without significance) (shown in Table 1).

The next issue is to find the underlying mechanism. SUA itself acts as an inflammatory cytokine and induces an oxidative status, consequently causing endothelial dysfunction (28). When UA is released from dead cells, it acts as a proinflammatory signal like other danger-associated molecular patterns (DAMPs) (4, 29). In addition, the process of nucleotide degradation from massive cell death to UA is also accompanied by the production of a large number of oxygen-free radicals (29). Therefore, we speculated that SUA could amplify the postoperative inflammatory response and ROS stress, which might be more obvious in patients with a proinflammatory background. The whole procedure subsequently causes damage to myocardial and cardiac vascular endothelial cells, and SUA acts as a trigger of the cascade. In addition, SUA generation causes the consumption of large amounts of adenosine triphosphate (ATP), which plays an important role in cardiac mechanical and electrical activities (30). The consumption of large amounts of ATP induces a disturbance in cardiac conduction and contraction, which consequently aggravates the damage caused by the inflammation cascade (31).

Although G4 had a lower average LVEF (56.5 ± 11.3%) than the other groups, our data did not reveal a significant correlation of baseline LVEF with adverse outcomes. The possible explanations may include the following: (1) we excluded patients with emergency surgery who were more likely to have a lower than normal baseline LVEF—the exclusion of these patients may have concealed the effect of LVEF—and (2) we only included preoperative LVEF, not LVEF variation, in our study. As a morphologic indicator, LVEF variation may also be the consequence of the inflammation cascade. However, SUA variation may not parallel the decrease in LVEF; therefore, a continuous record of LVEF after surgery may be crucial.

Finally, we proposed the SUA increase cutoff value (54.5 µmol/L for all-cause death and 42.6 µmol/L for fatal arrhythmia) in the overall population. We used the cutoff value to group the patients and the result showed G4 had the worst outcomes. However, the cutoff values varied in different subgroups, which is not convenient for clinical work (from 52.5 to 99.4 µmol/L for all-cause death and from 42.6 to 75.6 µmol/L for fatal arrhythmia). Therefore, we chose a fixed value (30 µmol/L) as the criterion to divide the four groups in the overall patients and the subgroups. Therefore, physicians may only pay attention to that if the increase in SUA reaches a certain value after surgery; they do not need to consider the characteristics (e.g., age and sex) of any particular patients. Advances in technology have made it possible to monitor the variation in perioperative SUA levels rapidly and non-invasively (32), and our results might provide physicians with some insights into the identification of patients potentially at risk.

The study has some limitations. First, it was a retrospective study; therefore, inherent limitations cannot be avoided. Second, we only included perioperative outcomes and provided limited information regarding long-term outcomes. However, we assume that an acute SUA increase may be more likely to correlate with short-term outcomes such as in-hospital all-cause death. Thus, our study design was quite reasonable. The third limitation was that we used EuroSCORE instead of EuroSCORE II due to incomplete information in our data. However, the role of EuroSCORE I in our study was to illustrate the fact the four groups had a similar preoperative risk. Although the (mortality) risk obtained with EuroSCORE I may be overestimated or underestimated (not as accurate as EuroSCORE II), we think this may not influence the result (the role of uric acid variation). The fourth limitation was that the peak value of postoperative SUA was acquired by selecting the highest value among several tests. The actual peak value might not be captured. Recently, new methods emerged that could measure SUA levels rapidly and even non-invasively (32). Continuous monitoring might be possible for future research. Next, the impact of certain drugs on SUA, such as diuretics, was not discussed in the study. However, few studies have illustrated the effect of certain kinds and dosages of diuretics, and heterogeneity made the issue more complex. We aimed to remind the physicians to pay attention when uric acid increases after surgery. It may be not the most important thing for us to illustrate the causes. Finally, we could not demonstrate the association between a decrease in perioperative SUA levels and adverse outcomes, as we could not differentiate a pathological decrease from a physiological decrease among patients with decreased SUA after surgery. In addition, there was no standard on pathological SUA decreases, as far as we know.

## Conclusion

Our study is the first known to propose that an acute SUA increase correlated with in-hospital adverse outcomes in a CABG population. Our data showed that a perioperative SUA level increase of ≥30 µmol/L was significantly associated with in-hospital all-cause death and fatal arrhythmia. This association was independent of baseline SUA and renal function and had some discrepancies in different subgroups. In addition, we proposed the SUA increase cutoff values for in-hospital all-cause death (54.5 µmol/L) and in-hospital fatal arrhythmia (42.6 µmol/L). In conclusion, perioperative SUA variation may have some value in the identification of potentially high-risk individuals among relatively low-risk patients.

## Data Availability

The raw data supporting the conclusions of this article will be made available by the authors, without undue reservation.
