# Supplementary material for: The association of perioperative serum uric acid variation with in-hospital adverse outcomes in coronary artery bypass grafting patients
Source: Front Cardiovasc Med. 2024 Oct 1;11:1364744. doi: 10.3389/fcvm.2024.1364744 (PMC11475021; doi:10.3389/fcvm.2024.1364744)
Supplement: Supplementary file 1 [file Table1.docx]

**Supplementary Table 1 Basic characteristics and incidence of adverse outcomes in patients ≥ 60 years.**

|  | **All (n=1338)** | **G1(n=547)** | **G2(n=622)** | **G3(n=88)** | **G4(n=81)** | ***P*-value** |
| --- | --- | --- | --- | --- | --- | --- |
| **General conditions** |  |  |  |  |  |  |
| Age (years) | 67.3±4.5 | 67.3±4.7 | 67.4±4.5 | 66.6±3.9 | 66.0±4.1 c,† | 0.032 |
| Male, (%) | 71.5 | 74.2 | 68.5 a | 76.1 | 71.6 | 0.128 |
| BMI (kg/m^2^) | 25.1±3.0 | 24.9±2.9 | 25.2±3.1 | 25.0±2.8 | 25.9±3.1 c,† | 0.047 |
| BMI ≥ 25 (kg/m^2^), (%) | 47.1 | 44.5 | 47.8 | 46.0 | 60.3 c,† | 0.071 |
| Smoking history, (%) | 45.3 | 46.1 | 44.9 | 46.6 | 42.0 | 0.896 |
| Drinking history, (%) | 28.8 | 31.4 | 25.4 b | 40.9 * | 23.5 ‡ | 0.005 |
| HTN history, (%) | 68.9 | 70.6 | 67.7 | 67.0 | 69.1 | 0.734 |
| DM history, (%) | 37.8 | 33.1 | 38.4 | 51.1 b,* | 50.6 c,† | 0.001 |
| Insulin, (%) | 8.6 | 7.9 | 8.7 | 10.2 | 11.1 | 0.722 |
| MI history, (%) | 24.3 | 24.7 | 24.0 | 20.5 | 28.4 | 0.676 |
| PCI history, (%) | 11.6 | 13.0 | 10.6 | 10.2 | 11.1 | 0.617 |
| Stroke/TIA history, (%) | 18.4 | 16.6 | 19.5 | 20.5 | 19.8 | 0.585 |
| AF history, (%) | 3.4 | 3.5 | 2.9 | 5.7 | 3.7 | 0.636 |
| COPD history, (%) | 0.5 | 0.4 | 0.3 | 1.1 | 2.5 | 0.220 |
| Euro SCORE I ≥ 6, (%) | 6.2 | 6.6 | 6.3 | 2.3 | 7.4 | 0.464 |
| **Preoperative and perioperative conditions** | | |  |  |  |  |
| TC (mmol/L) | 4.07±1.03 | 4.05±1.11 | 4.09±0.99 | 4.08±0.95 | 4.14±0.91 | 0.844 |
| TG (mmol/L) | 1.29[0.94,1.78] | 1.28[0.94,1.75] | 1.27[0.92,1.76] | 1.34[1.01,1.84] | 1.47[1.08,2.09] | 0.384 |
| HDL-C (mmol/L) | 1.05±0.24 | 1.05±0.24 | 1.05±0.25 | 1.03±0.20 | 1.01±0.23 | 0.491 |
| LDL-C (mmol/L) | 2.44±0.86 | 2.42±0.92 | 2.45±0.84 | 2.44±0.77 | 2.55±0.76 | 0.651 |
| CREA (μmol/L) | 73.0±14.1 | 76.0±14.3 | 71.1±13.8 a | 70.5±13.2 b | 69.9±12.5 c | <0.001 |
| eGFR (mL/min/1.73 m^2^) | 87.2±11.0 | 85.1±11.3 | 88.2±10.7 a | 89.9±9.8 b | 90.6±9.7 c | <0.001 |
| eGFR ≥ 90 (mL/min/1.73 m^2^), (%) | 49.1 | 41.1 | 52.5 a | 63.6 b,* | 60.5 c | <0.001 |
| SUA (μmol/L) | 317.8±82.4 | 361.4±75.7 | 293.7±70.4 | 267.6±79.0 b,* | 262.7±76.2 c,† | <0.001 |
| HUA (%) | 13.9 | 24.7 | 6.9 a | 4.5 b | 4.9 c | <0.001 |
| Na^+^(mmol/L) | 140.387±2.498 | 140.381±2.340 | 140.361±2.661 | 140.955±2.227 b,* | 140.010±2.460 ‡ | 0.091 |
| Cl^-^ (mmol/L) | 102.561±2.921 | 102.559±2.868 | 102.548±2.989 | 102.853±2.660 | 102.359±3.038 | 0.734 |
| K^+^ (mmol/L) | 4.137±0.364 | 4.126±0.354 | 4.153±0.371 | 4.078±0.378 | 4.154±0.361 | 0.243 |
| Mg^2+^(mmol/L) | 0.908±0.078 | 0.907±0.078 | 0.909±0.079 | 0.903±0.073 | 0.901±0.074 | 0.776 |
| Ca^2+^(mmol/L) | 2.341±0.111 | 2.341±0.115 | 2.342±0.109 | 2.342±0.112 | 2.334±0.098 | 0.925 |
| hs-CRP(mg/L) | 1.43[0.61,3.99] | 1.41[0.61,3.67] | 1.50[0.59,4.03] | 1.25[0.68,5.80] | 1.87[0.76,4.45] | 0.515 |
| LVEF (%) | 60.9±8.7 | 61.1±8.6 | 60.9±8.8 | 61.8±8.3 | 59.2±10.0 | 0.231 |
| LVEF ≥ 50%, (%) | 89.8 | 89.7 | 90.8 | 90.7 | 81.5 c,† | 0.078 |
| Preoperative heart rate(bpm) | 75.3±10.3 | 74.8±10.7 | 75.7±9.9 | 76.2±10.9 | 74.8±10.0 | 0.367 |
| Preoperative PR (ms) | 164.0±26.4 | 164.9±27.6 | 162.9±25.2 | 163.3±28.1 | 166.2±24.6 | 0.523 |
| Preoperative QTc (ms) | 435.4±35.0 | 435.8±35.8 | 435.3±34.9 | 430.9±31.2 | 437.4±35.1 | 0.617 |
| Preoperative SBP (mmHg) | 129.7±16.4 | 127.5±15.7 | 130.5±16.7 a | 132.1±16.6 b | 135.8±16.0 c,† | <0.001 |
| Preoperative DBP (mmHg) | 74.7±9.7 | 73.8±9.7 | 75.5±9.7 a | 74.3±9.1 | 74.1±9.7 | 0.032 |
| Blood Loss(ml) | 700[600,900] | 700[600,900] | 700[500,900] | 800[500,1000] | 800[600,1000] | 0.777 |
| **Postoperative conditions** |  |  |  |  |  |  |
| CREA (μmol/L) | 77.0±16.9 | 75.4±17.3 | 77.2±16.7 | 79.0±16.8 | 83.9±12.6 c,† | <0.001 |
| eGFR (mL/min/1.73 m^2^) | 82.9±13.6 | 84.3±12.9 | 82.5±13.2 a | 82.2±13.5 | 77.6±11.3 c,†,‡ | <0.001 |
| SUA (μmol/L) | 242.6±74.1 | 221.7±63.3 | 243.0±69.2 a | 281.2±78.7 b | 338.9±79.9 c,†,‡ | <0.001 |
| Na^+^(mmol/L) | 140.023±4.022 | 139.706±3.907 | 140.062±3.953 | 141.127±4.756 b,* | 140.624±4.199 | 0.008 |
| Cl^-^ (mmol/L) | 104.759±3.476 | 104.783±3.567 | 104.727±3.377 | 105.119±3.621 | 104.453±3.478 | 0.649 |
| K^+^ (mmol/L) | 4.134±0.372 | 4.146±0.359 | 4.127±0.377 | 4.134±0.445 | 4.112±0.335 | 0.774 |
| Mg^2+^(mmol/L) | 0.836±0.129 | 0.834±0.131 | 0.839±0.131 | 0.831±0.102 | 0.826±0.126 | 0.826 |
| Ca^2+^(mmol/L) | 1.986±0.154 | 1.980±0.157 | 1.990±0.155 | 1.980±0.147 | 1.997±0.143 | 0.636 |
| **In-hospital adverse outcomes** |  |  |  |  |  |  |
| Fatal arrhythmia, (%) | 1.6 | 1.3 | 1.4 | 0 | 6.2 c,†,‡ | 0.023 |
| All-cause death, (%) | 1.0 | 0.7 | 1.0 | 0 | 4.9 c,†,‡ | 0.033 |

**Group 1 (G1)** and **Group 2 (G2)** had perioperative SUA decrease of ≥ 90 µmol/L and < 90 µmol/L respectively. **Group 3 (G3)** and **Group 4 (G4)** had perioperative SUA increase of < 30 µmol/L and ≥ 30 µmol/L respectively. **Abbreviations:** BMI: Body mass index; HTN: Hypertension; DM: Diabetes mellitus; MI: Myocardial infarction; PCI: Percutaneous coronary intervention; TIA: Transient ischemic attack; AF: Atrial fibrillation; COPD: Chronic obstructive pulmonary disease; EuroSCORE: European System for Cardiac Operative Risk Evaluation; TG: Triglyceride; TCHO: Total cholesterol; HDL-C: High density lipoprotein cholesterol; LDL-C: Low density lipoprotein cholesterol; CREA: Creatinine; eGFR: estimated glomerular filtration rate; SUA: Serum uric acid; HUA: high preoperative serum uric acid; hsCRP: high sensitive c-reactive protein; LVEF: Left ventricular ejection fraction; PR:P-R interval; QTc: Q-T corrected interval; SBP: Systolic blood pressure; DBP: Diastolic blood pressure. **Continuous variables were described as mean ± standard deviation or median with interquartile range, as appropriate. Categorical variables were described as percentages (%). The character “a”** represents a significant difference between G1 and G2; **The character “b”** represents a significant difference between G1 and G3; **The character “c”** represents a significant difference between G1 and G4; **The character “*”** represents a significant difference between G2 and G3; **The character “†”** represents a significant difference between G2 and G4; **The character “‡”** represents a significant difference between G3 and G4.

**Supplementary Table 2 Basic characteristics and incidence of adverse outcomes in patients < 60 years.**

|  | **All (n=1115)** | **G1(n=483)** | **G2(n=472)** | **G3(n=75)** | **G4(n=85)** | ***P*-value** |
| --- | --- | --- | --- | --- | --- | --- |
| **General conditions** |  |  |  |  |  |  |
| Age (years) | 53.2±5.7 | 53.1±5.8 | 53.3±5.2 | 53.6±6.4 | 53.3±6.8 | 0.900 |
| Male, (%) | 83.0 | 86.1 | 80.1 a | 77.3 b | 85.9 | 0.039 |
| BMI (kg/m^2^) | 25.9±3.0 | 25.8±3.0 | 25.8±2.9 | 26.3±3.4 | 26.9±3.5 c,† | 0.008 |
| BMI ≥ 25 (kg/m^2^), (%) | 59.4 | 58.7 | 57.4 | 62.7 | 71.1 c,† | 0.117 |
| Smoking history, (%) | 59.6 | 57.0 | 60.2 | 62.7 | 64.7 | 0.549 |
| Drinking history, (%) | 28.6 | 30.6 | 26.3 | 29.3 | 29.4 | 0.515 |
| HTN history, (%) | 60.6 | 63.6 | 57.4 | 65.3 | 57.6 | 0.188 |
| DM history, (%) | 37.0 | 30.6 | 43.0 a | 34.7 | 42.4 c | 0.001 |
| Insulin, (%) | 9.2 | 7.2 | 11.4 a | 8.0 | 9.4 | 0.161 |
| MI history, (%) | 32.8 | 30.8 | 33.9 | 32.0 | 38.8 | 0.473 |
| PCI history, (%) | 14.2 | 15.3 | 12.9 | 14.7 | 14.1 | 0.766 |
| Stroke/TIA history, (%) | 11.2 | 12.4 | 9.7 | 16.0 | 8.2 | 0.239 |
| AF history, (%) | 1.3 | 1.2 | 1.5 | 0 | 1.2 | 0.555 |
| COPD history, (%) | 0.2 | 0.2 | 0.2 | 0 | 0 | 0.892 |
| Euro SCORE I ≥ 6, (%) | 0 | 0 | 0 | 0 | 0 |  |
| **Preoperative and perioperative conditions** | | |  |  |  |  |
| TC (mmol/L) | 4.24±1.18 | 4.16±1.10 | 4.30±1.23 | 4.16±1.16 | 4.43±1.33 | 0.134 |
| TG (mmol/L) | 1.53[1.11,2.20] | 1.52[1.10,2.21] | 1.52[1.10,2.16] | 1.46[1.13,2.35] | 1.60[1.26,2.23] | 0.196 |
| HDL-C (mmol/L) | 0.96±0.23 | 0.96±0.25 | 0.96±0.23 | 0.94±0.18 | 0.94±0.21 | 0.681 |
| LDL-C (mmol/L) | 2.61±1.01 | 2.53±0.94 | 2.68±1.09 a | 2.44±0.84 | 2.81±1.07 c,‡ | 0.012 |
| CREA (μmol/L) | 73.1±13.8 | 74.9±13.7 | 71.4±14.0 a | 71.4±12.9 b | 73.4±14.3 | 0.001 |
| eGFR (mL/min/1.73 m^2^) | 97.8±11.8 | 96.7±12.1 | 98.8±11.3 a | 97.8±12.5 | 98.2±11.7 | 0.051 |
| eGFR ≥ 90 (mL/min/1.73 m^2^), (%) | 78.5 | 75.7 | 81.4 a | 77.3 | 80.0 | 0.199 |
| SUA (μmol/L) | 333.3±83.4 | 366.9±78.6 | 311.4±76.7 a | 291.6±80.7 b,* | 300.9±79.1 c | <0.001 |
| HUA (%) | 15.3 | 24.4 | 8.7 a | 8.0 b | 7.1 c | <0.001 |
| Na^+^(mmol/L) | 140.360±2.416 | 140.406±2.143 | 140.289±2.571 | 140.229±2.791 | 140.615±2.644 | 0.626 |
| Cl^-^ (mmol/L) | 102.487±2.870 | 102.551±2.621 | 102.420±2.961 | 102.225±3.034 | 102.731±3.518 | 0.630 |
| K^+^ (mmol/L) | 4.109±0.343 | 4.098±0.337 | 4.128±0.342 | 4.052±0.348 | 4.115±0.380 | 0.263 |
| Mg^2+^(mmol/L) | 0.898±0.079 | 0.900±0.078 | 0.897±0.078 | 0.889±0.078 | 0.893±0.093 | 0.636 |
| Ca^2+^(mmol/L) | 2.350±0.107 | 2.358±0.107 | 2.343±0.108 a | 2.348±0.808 | 2.347±0.117 | 0.193 |
| hs-CRP(mg/L) | 1.61[0.64,4.18] | 1.53[0.57,4.15] | 1.67[0.65,4.26] | 1.85[0.83,3.81] | 1.55[0.70,4.38] | 0.211 |
| LVEF (%) | 59.4±9.8 | 60.3±9.2 | 59.5±9.7 | 59.8±8.9 | 54.0±11.9 c,†,‡ | <0.001 |
| LVEF ≥ 50%, (%) | 86.1 | 87.9 | 87.6 | 89.2 | 64.7 c,†,‡ | <0.001 |
| Preoperative heart rate(bpm) | 75.6±10.2 | 75.8±10.3 | 75.6±10.3 | 75.0±8.3 | 75.5±9.7 | 0.922 |
| Preoperative PR (ms) | 162.4±25.6 | 160.9±24.1 | 162.8±25.9 | 166.1±26.2 | 164.8±30.5 | 0.267 |
| Preoperative QTc (ms) | 431.9±33.4 | 433.0±33.5 | 430.3±32.9 | 437.7±35.1 | 430.0±33.9 | 0.249 |
| Preoperative SBP (mmHg) | 127.6±15.7 | 127.0±15.0 | 126.6±15.8 | 132.3±15.6 b,* | 131.9±17.7 c,† | 0.001 |
| Preoperative DBP (mmHg) | 75.4±9.8 | 75.3±10.1 | 75.3±9.6 | 75.4±9.7 | 75.7±10.0 | 0.993 |
| Blood Loss(ml) | 700[600,1000] | 700[600,1000] | 700[600,900] | 800[600,900] | 800[600,1000] | 0.112 |
| **Postoperative conditions** |  |  |  |  |  |  |
| CREA (μmol/L) | 75.6±17.0 | 73.3±17.7 | 75.8±16.5 a | 79.8±13.6 b,* | 83.6±14.8 c,† | <0.001 |
| eGFR (mL/min/1.73 m^2^) | 94.5±14.3 | 96.8±14.0 | 94.2±14.0 a | 90.3±13.2 b,* | 87.6±15.1 c,† | <0.001 |
| SUA (μmol/L) | 258.2±86.3 | 224.3±68.3 | 263.7±78.2 a | 303.7±81.0 b,* | 389.6±90.1 c | <0.001 |
| Na^+^(mmol/L) | 139.793±3.935 | 139.449±3.613 | 139.908±3.947 | 139.773±4.559 | 141.118±4.714 c,†,‡ | 0.003 |
| Cl^-^ (mmol/L) | 104.355±3.574 | 104.071±3.434 | 104.561±3.381 a | 104.127±4.265 | 105.033±4.499 c | 0.047 |
| K^+^ (mmol/L) | 4.153±0.378 | 4.150±0.373 | 4.155±0.383 | 4.200±0.330 | 4.119±0.413 | 0.598 |
| Mg^2+^(mmol/L) | 0.833±0.133 | 0.835±0.135 | 0.838±0.131 | 0.794±0.138 b,* | 0.828±0.128 | 0.078 |
| Ca^2+^(mmol/L) | 2.001±0.143 | 2.006±0.141 | 1.997±0.141 | 2.011±0.134 | 1.980±0.168 | 0.365 |
| **In-hospital adverse outcomes** |  |  |  |  |  |  |
| Fatal arrhythmia, (%) | 1.7 | 1.2 | 1.7 | 1.3 | 4.7 c | 0.278 |
| All-cause death, (%) | 0.9 | 1.2 | 0.6 | 1.3 | 0 | 0.448 |

**Supplementary Table 3 Basic characteristics and incidence of adverse outcomes in male patients.**

|  | **All (n=1882)** | **G1(n=822)** | **G2(n=804)** | **G3(n=125)** | **G4(n=131)** | ***P*-value** |
| --- | --- | --- | --- | --- | --- | --- |
| **General conditions** |  |  |  |  |  |  |
| Age (years) | 60.1±8.8 | 59.9±9.0 | 60.5±8.6 | 60.2±8.4 | 58.3±8.7 † | 0.058 |
| Age ≥ 60 (years), (%) | 50.9 | 49.4 | 53.0 | 53.6 | 44.3 | 0.186 |
| BMI (kg/m2) | 25.6±2.9 | 25.4±2.8 | 25.6±3.0 | 25.5±2.8 | 26.4±3.5 c,†,‡ | 0.003 |
| BMI ≥ 25 (kg/m^2^), (%) | 53.9 | 52.8 | 53.0 | 55.6 | 64.1 c,† | 0.106 |
| Smoking history, (%) | 64.3 | 62.5 | 65.9 | 65.6 | 64.9 | 0.541 |
| Drinking history, (%) | 33.5 | 35.2 | 31.2 | 42.4 * | 28.2 ‡ | 0.030 |
| HTN history, (%) | 63.0 | 65.6 | 60.4 a | 64.8 | 60.3 | 0.161 |
| DM history, (%) | 35.4 | 30.5 | 38.3 a | 41.6 b | 42.0 c | 0.001 |
| Insulin, (%) | 8.7 | 6.7 | 10.3 a | 8.8 | 11.5 | 0.044 |
| MI history, (%) | 30.2 | 28.7 | 31.3 | 26.4 | 35.9 | 0.238 |
| PCI history, (%) | 13.0 | 13.6 | 12.3 | 12.8 | 13.0 | 0.891 |
| Stroke/TIA history, (%) | 14.6 | 14.4 | 13.9 | 20.0 | 14.5 | 0.354 |
| AF history, (%) | 2.3 | 2.6 | 2.1 | 4.0 | 0.8 | 0.319 |
| COPD history, (%) | 0.4 | 0.4 | 0.4 | 0 | 0.8 | 0.718 |
| Euro SCORE I ≥ 6, (%) | 2.2 | 2.2 | 2.8 | 0 | 0.8 | 0.159 |
| **Preoperative and perioperative conditions** | | |  |  |  |  |
| TC (mmol/L) | 4.05±1.05 | 3.98±1.02 | 4.09±1.07 a | 4.03±1.06 | 4.20±1.11 c | 0.059 |
| TG (mmol/L) | 1.36[0.99,1.91] | 1.36[1.00,1.91] | 1.33[0.95,1.88] | 1.40[1.05,2.04] | 1.50[1.11,2.17] | 0.306 |
| HDL-C (mmol/L) | 0.98±0.22 | 0.98±0.22 | 0.99±0.23 | 0.97±0.19 | 0.95±0.22 | 0.257 |
| LDL-C (mmol/L) | 2.46±0.90 | 2.39±0.87 | 2.52±0.95 a | 2.39±0.77 | 2.63±0.90 c,‡ | 0.003 |
| CREA (μmol/L) | 76.8±12.7 | 78.8±12.8 | 75.5±12.5 a | 74.3±12.1 b | 74.7±12.2 c | <0.001 |
| eGFR (mL/min/1.73 m2) | 92.6±12.7 | 91.1±13.2 | 93.4±12.3 a | 94.1±12.2 b | 95.7±11.6 c | <0.001 |
| eGFR ≥ 90 (mL/min/1.73 m^2^), (%) | 64.0 | 58.6 | 66.7 a | 71.2 b | 74.0 c | <0.001 |
| SUA (μmol/L) | 337.0±81.1 | 371.0±76.4 | 316.5±71.7 a | 293.0±79.7 b,* | 291.3±80.9 c,† | <0.001 |
| HUA (%) | 14.5 | 23.0 | 8.5 a | 6.4 b | 5.3 c | <0.001 |
| Na^+^(mmol/L) | 140.349±2.452 | 140.317±2.255 | 140.321±2.649 | 140.566±2.413 | 140.509±2.433 | 0.620 |
| Cl^-^ (mmol/L) | 102.479±2.916 | 102.470±2.773 | 102.437±3.034 | 102.513±2.728 | 102.761±3.234 | 0.702 |
| K^+^ (mmol/L) | 4.129±0.354 | 4.111±0.345 | 4.156±0.362 a | 4.053±0.354 * | 4.145±0.351 ‡ | 0.005 |
| Mg^2+^(mmol/L) | 0.900±0.078 | 0.900±0.076 | 0.903±0.079 | 0.893±0.076 | 0.892±0.082 | 0.326 |
| Ca^2+^(mmol/L) | 2.343±0.107 | 2.346±0.109 | 2.341±0.107 | 2.341±0.096 | 2.337±0.107 | 0.753 |
| hs-CRP(mg/L) | 1.54[0.62,3.99] | 1.45[0.56,3.73] | 1.59[0.61,4.13] | 1.34[0.77,5.20] | 1.86[0.76,4.46] | 0.053 |
| LVEF (%) | 59.6±9.4 | 60.4±9.0 | 59.4±9.4 a | 60.4±8.9 | 55.1±10.3 c,†,‡ | <0.001 |
| LVEF ≥ 50%, (%) | 86.9 | 88.4 | 88.1 | 88.6 | 68.7 c,†,‡ | <0.001 |
| Preoperative heart rate(bpm) | 75.4±10.1 | 75.1±10.5 | 75.6±10.1 | 75.6±9.4 | 75.2±8.7 | 0.832 |
| Preoperative PR (ms) | 164.4±25.8 | 163.5±24.4 | 164.7±26.4 | 165.8±28.1 | 166.7±28.4 | 0.470 |
| Preoperative QTc (ms) | 432.4±33.6 | 432.8±33.0 | 432.0±34.2 | 432.0±33.9 | 433.6±33.9 | 0.943 |
| Preoperative SBP (mmHg) | 128.9±15.9 | 127.6±15.4 | 129.0±16.1 | 131.9±15.3 b | 133.1±17.3 c,† | <0.001 |
| Preoperative DBP (mmHg) | 75.3±9.9 | 74.9±10.0 | 75.7±9.7 | 75.7±9.7 | 75.1±10.3 | 0.388 |
| Blood Loss(ml) | 700[600,1000] | 800[600,1000] | 700[550,900] | 800[600,1000] | 800[600,1000] | 0.637 |
| **Postoperative conditions** |  |  |  |  |  |  |
| CREA (μmol/L) | 80.0±16.1 | 77.5±17.1 | 81.1±15.3 a | 83.1±13.9 b | 86.2±13.3 c,† | <0.001 |
| eGFR (mL/min/1.73 m^2^) | 88.9±14.6 | 90.7±14.7 | 88.1±14.5 a | 86.7±13.4 b | 84.9±13.9 c,† | <0.001 |
| SUA (μmol/L) | 260.1±80.8 | 228.6±66.2 | 267.3±72.1 a | 306.0±79.5 b,* | 370.5±88.2 c,†,‡ | <0.001 |
| Na^+^(mmol/L) | 139.802±3.838 | 139.479±3.643 | 139.874±3.808 a | 140.212±4.548 b | 140.970±4.202 c,† | <0.001 |
| Cl^-^ (mmol/L) | 104.407±3.435 | 104.280±3.451 | 104.447±3.267 | 104.517±3.798 | 104.850±3.929 | 0.317 |
| K^+^ (mmol/L) | 4.152±0.372 | 4.159±0.363 | 4.148±0.372 | 4.177±0.406 | 4.111±0.394 | 0.462 |
| Mg^2+^(mmol/L) | 0.832±0.132 | 0.831±0.136 | 0.837±0.129 | 0.807±0.122 * | 0.827±0.127 | 0.123 |
| Ca^2+^(mmol/L) | 1.991±0.144 | 1.991±0.147 | 1.990±0.141 | 1.991±0.137 | 1.995±0.148 | 0.991 |
| **In-hospital adverse outcomes** |  |  |  |  |  |  |
| Fatal arrhythmia, (%) | 1.5 | 1.3 | 1.5 | 0 | 4.6 ‡ | 0.025 |
| All-cause death, (%) | 1.0 | 1.1 | 1.0 | 0 | 1.5 | 0.407 |

**Supplementary Table 4 Basic characteristics and incidence of adverse outcomes in female patients.**

|  | **All (n=571)** | **G1(n=208)** | **G2(n=290)** | **G3(n=38)** | **G4(n=35)** | ***P*-value** |
| --- | --- | --- | --- | --- | --- | --- |
| **General conditions** |  |  |  |  |  |  |
| Age (years) | 63.6±7.6 | 63.8±7.5 | 63.6±7.9 | 62.0±7.8 | 63.8±6.1 | 0.585 |
| Age ≥ 60 (years), (%) | 66.7 | 67.8 | 67.6 | 55.3 | 65.7 | 0.481 |
| BMI (kg/m2) | 25.1±3.4 | 24.9±3.5 | 24.9±3.2 | 26.0±4.0 | 26.3±2.6 c,† | 0.043 |
| BMI ≥ 25 (kg/m^2^), (%) | 48.8 | 44.9 | 49.0 | 47.4 | 72.7 c,†,‡ | 0.032 |
| Smoking history, (%) | 10.3 | 7.7 | 11.4 | 15.8 | 11.4 | 0.369 |
| Drinking history, (%) | 13.0 | 13.9 | 10.7 | 13.2 | 20.0 | 0.331 |
| HTN history, (%) | 72.3 | 74.0 | 71.0 | 71.1 | 74.3 | 0.886 |
| DM history, (%) | 44.3 | 37.5 | 46.2 | 50.0 | 62.9 c | 0.021 |
| Insulin, (%) | 9.5 | 11.1 | 8.6 | 10.5 | 5.7 | 0.672 |
| MI history, (%) | 21.5 | 23.1 | 19.7 | 23.7 | 25.7 | 0.714 |
| PCI history, (%) | 12.1 | 15.9 | 9.7 a | 10.5 | 11.4 | 0.221 |
| Stroke/TIA history, (%) | 17.0 | 15.9 | 19.0 | 13.2 | 11.4 | 0.541 |
| AF history, (%) | 2.6 | 1.9 | 2.8 | 0 | 8.6 c | 0.136 |
| COPD history, (%) | 0.4 | 0 | 0 | 2.6 | 2.9 | 0.041 |
| Euro SCORE I ≥ 6, (%) | 7.4 | 8.7 | 5.9 | 5.4 | 14.3 | 0.300 |
| **Preoperative and perioperative conditions** | | |  |  |  |  |
| TC (mmol/L) | 4.49±1.20 | 4.58±1.29 | 4.42±1.15 | 4.39±0.96 | 4.62±1.25 | 0.401 |
| TG (mmol/L) | 1.43[1.11,2.06] | 1.49[1.08,1.95] | 1.38[1.12,2.07] | 1.46[1.19,2.18] | 1.52[1.26,2.20] | 0.555 |
| HDL-C (mmol/L) | 1.09±0.28 | 1.10±0.31 | 1.08±0.28 | 1.07±0.20 | 1.05±0.23 | 0.620 |
| LDL-C (mmol/L) | 2.71±1.03 | 2.80±1.09 | 2.63±0.99 | 2.61±0.89 | 2.90±1.03 | 0.184 |
| CREA (μmol/L) | 60.5±10.1 | 62.2±10.1 | 59.4±10.2 a | 59.8±9.2 | 60.3±8.9 | 0.019 |
| eGFR (mL/min/1.73 m^2^) | 89.9±11.8 | 88.2±12.3 | 90.8±11.8 a | 91.7±10.3 | 90.1±9.6 | 0.070 |
| eGFR ≥ 90 (mL/min/1.73 m^2^), (%) | 57.4 | 52.4 | 60.0 | 65.8 | 57.1 | 0.259 |
| SUA (μmol/L) | 284.9±77.5 | 336.4±73.8 | 259.3±61.8 a | 231.5±63.9 b,* | 248.7±66.1 c | <0.001 |
| HUA (%) | 14.9 | 30.8 | 5.5 a | 5.3 b | 8.6 c | <0.001 |
| Na^+^(mmol/L) | 140.461±2.488 | 140.690±2.203 | 140.354±2.550 | 140.803±2.874 | 139.611±2.945 c,‡ | 0.069 |
| Cl^-^ (mmol/L) | 102.687±2.830 | 102.892±2.657 | 102.647±2.809 | 102.734±3.241 | 101.757±3.411 c | 0.177 |
| K^+^ (mmol/L) | 4.109±0.357 | 4.122±0.351 | 4.102±0.346 | 4.109±0.394 | 4.093±0.439 | 0.934 |
| Mg^2+^(mmol/L) | 0.912±0.081 | 0.918±0.085 | 0.908±0.078 | 0.908±0.075 | 0.917±0.090 | 0.608 |
| Ca^2+^(mmol/L) | 2.353±0.115 | 2.361±0.121 | 2.346±0.113 | 2.356±0.107 | 2.356±0.111 | 0.559 |
| hs-CRP(mg/L) | 1.51[0.63,4.23] | 1.48[0.63,4.31] | 1.60[0.59,3.94] | 1.50[0.67,5.73] | 1.13[0.63,4.21] | 0.803 |
| LVEF (%) | 62.4±8.4 | 62.0±8.4 | 62.8±8.4 | 62.2±7.4 | 61.7±9.9 | 0.689 |
| LVEF ≥ 50%, (%) | 92.0 | 90.8 | 93.0 | 94.6 | 88.6 | 0.635 |
| Preoperative heart rate(bpm) | 75.7±10.6 | 75.6±10.8 | 75.8±10.1 | 75.7±11.1 | 74.9±13.4 | 0.970 |
| Preoperative PR (ms) | 159.6±26.4 | 161.6±32.0 | 157.9±22.1 | 160.6±23.9 | 161.0±25.4 | 0.486 |
| Preoperative QTc (ms) | 438.3±36.3 | 441.3±40.4 | 436.4±33.8 | 440.9±29.7 | 433.7±37.5 | 0.392 |
| Preoperative SBP (mmHg) | 128.4±16.7 | 126.2±15.3 | 128.4±17.3 | 132.9±18.4 b | 136.3±15.7 c,† | 0.003 |
| Preoperative DBP (mmHg) | 73.9±9.3 | 73.2±9.2 | 74.7±9.6 | 72.0±7.8 | 74.3±8.0 | 0.191 |
| Blood Loss(ml) | 700[600,900] | 600[550,800] | 700[600,900] | 800[600,1000] | 800[600,1000] | 0.209 |
| **Postoperative conditions** |  |  |  |  |  |  |
| CREA (μmol/L) | 64.2±13.6 | 62.1±13.3 | 64.1±13.4 | 67.1±13.7 b | 74.7±11.3 c,†,‡ | <0.001 |
| eGFR (mL/min/1.73 m^2^) | 85.8±15.2 | 87.8±14.7 | 86.0±15.2 | 83.3±15.4 | 74.6±12.8 c,†,‡ | <0.001 |
| SUA (μmol/L) | 215.3±67.6 | 200.4±58.5 | 209.3±61.0 | 244.2±64.0 b,* | 321.6±73.9 c,†,‡ | <0.001 |
| Na^+^(mmol/L) | 140.302±4.411 | 140.000±4.223 | 140.334±4.308 | 141.511±5.125 | 140.529±5.386 | 0.279 |
| Cl^-^ (mmol/L) | 105.128±3.761 | 105.102±3.723 | 105.234±3.614 | 105.170±4.433 | 104.374±4.434 | 0.650 |
| K^+^ (mmol/L) | 4.112±0.381 | 4.103±0.373 | 4.114±0.399 | 4.121±0.365 | 4.134±0.305 | 0.966 |
| Mg^2+^(mmol/L) | 0.843±0.128 | 0.849±0.120 | 0.841±0.136 | 0.841±0.114 | 0.826±0.127 | 0.782 |
| Ca^2+^(mmol/L) | 1.997±0.166 | 1.997±0.159 | 1.999±0.169 | 2.008±0.158 | 1.965±0.183 | 0.697 |
| **In-hospital adverse outcomes** |  |  |  |  |  |  |
| Fatal arrhythmia, (%) | 1.9 | 1.0 | 1.7 | 2.6 | 8.6 c,† | 0.118 |
| All-cause death, (%) | 0.9 | 0.5 | 0.3 | 2.6 | 5.7 c,† | 0.080 |

**Supplementary Table 5 Basic characteristics and incidence of adverse outcomes in patients with BMI** **≥ 25 kg/m^2^.**

|  | **All (n=1274)** | **G1(n=519)** | **G2(n=562)** | **G3(n=87)** | **G4(n=106)** | ***P*-value** |
| --- | --- | --- | --- | --- | --- | --- |
| **General conditions** |  |  |  |  |  |  |
| Age (years) | 59.7±8.9 | 59.3±9.0 | 60.3±8.8 a | 59.5±8.4 | 58.5±8.8 † | 0.100 |
| Age ≥ 60 (years), (%) | 48.7 | 46.2 | 52.3 a | 46.0 | 44.3 | 0.154 |
| Male, (%) | 78.3 | 82.1 | 74.9 a | 79.3 | 77.4 | 0.041 |
| BMI (kg/m^2^) | 27.7±2.2 | 27.6±2.1 | 27.7±2.2 | 27.8±2.4 | 28.1±2.8 c | 0.183 |
| Smoking history, (%) | 52.7 | 52.4 | 52.7 | 58.6 | 49.1 | 0.614 |
| Drinking history, (%) | 28.9 | 32.9 | 24.9 a | 33.3 | 26.4 | 0.022 |
| HTN history, (%) | 70.6 | 72.1 | 71.0 | 67.8 | 64.2 | 0.387 |
| DM history, (%) | 38.4 | 32.2 | 41.5 a | 41.4 | 50 c | 0.001 |
| Insulin, (%) | 9.4 | 7.7 | 10.9 | 9.2 | 10.4 | 0.353 |
| MI history, (%) | 28.6 | 26.6 | 29.7 | 31.0 | 31.1 | 0.583 |
| PCI history, (%) | 14.1 | 16.2 | 12.3 | 14.9 | 13.2 | 0.318 |
| Stroke/TIA history, (%) | 15.1 | 14.6 | 16.9 | 12.6 | 10.4 | 0.290 |
| AF history, (%) | 2.8 | 2.9 | 2.7 | 4.6 | 1.9 | 0.733 |
| COPD history, (%) | 0.3 | 0 | 0.2 | 1.1 b | 1.9 c,† | 0.034 |
| Euro SCORE I ≥ 6, (%) | 2.8 | 2.7 | 2.9 | 1.2 | 3.8 | 0.698 |
| **Preoperative and perioperative conditions** | | |  |  |  |  |
| TC (mmol/L) | 4.18±1.12 | 4.15±1.18 | 4.18±1.08 | 4.23±1.04 | 4.26±1.11 | 0.800 |
| TG (mmol/L) | 1.53[1.13,2.16] | 1.52[1.12,2.10] | 1.50[1.11,2.13] | 1.63[1.13,2.31] | 1.65[1.22,2.32] | 0.219 |
| HDL-C (mmol/L) | 0.97±0.21 | 0.96±0.21 | 0.98±0.23 | 0.95±0.`8 | 0.94±0.20 | 0.141 |
| LDL-C (mmol/L) | 2.55±0.94 | 2.53±0.98 | 2.55±0.93 | 2.52±0.78 | 2.66±0.87 | 0.589 |
| CREA (μmol/L) | 73.7±14.0 | 76.2±13.8 | 71.9±14.2 a | 72.9±12.2 b | 71.3±13.6 c | <0.001 |
| eGFR (mL/min/1.73 m^2^) | 92.4±12.7 | 91.1±13.4 | 93.0±12.3 a | 92.8±11.5 | 95.2±12.2 c | 0.007 |
| eGFR ≥ 90 (mL/min/1.73 m^2^), (%) | 63.8 | 58.8 | 66.0 a | 67.8 | 73.6 c | 0.008 |
| SUA (μmol/L) | 335.8±85.2 | 375.1±79.5 | 315.0±75.6 a | 293.0±86.5 b,* | 288.3±79.9 c,† | <0.001 |
| HUA (%) | 18.0 | 29.1 | 10.7 a | 10.3 b | 8.5 c | <0.001 |
| Na^+^(mmol/L) | 140.385±2.497 | 140.374±2.332 | 140.354±2.584 | 140.687±2.455 | 140.348±2.846 | 0.708 |
| Cl^-^ (mmol/L) | 102.621±2.916 | 102.757±2.781 | 102.510±2.914 | 102.681±2.768 | 102.498±3.622 | 0.539 |
| K^+^ (mmol/L) | 4.112±0.348 | 4.095±0.335 | 4.135±0.353 | 4.047±0.349 * | 4.132±0.376 | 0.072 |
| Mg^2+^(mmol/L) | 0.895±0.077 | 0.896±0.077 | 0.897±0.077 | 0.882±0.073 | 0.890±0.082 | 0.338 |
| Ca^2+^(mmol/L) | 2.341±0.109 | 2.342±0.113 | 2.342±0.108 | 2.336±0.087 | 2.342±0.107 | 0.970 |
| hs-CRP(mg/L) | 1.80[0.76,4.49] | 1.79[0.72,4.25] | 1.80[0.78,4.38] | 2.04[0.98,6.24] | 1.94[0.79,7.77] | 0.234 |
| LVEF (%) | 60.8±8.9 | 61.4±8.4 | 61.0±8.9 | 60.9±8.2 | 57.1±10.8 c,†,‡ | <0.001 |
| LVEF ≥ 50%, (%) | 90.3 | 91.8 | 91.8 | 90.7 | 74.5 c,†,‡ | <0.001 |
| Preoperative heart rate(bpm) | 75.4±10.4 | 75.3±10.4 | 75.7±10.5 | 74.2±9.2 | 75.2±10.4 | 0.663 |
| Preoperative PR (ms) | 164.1±26.5 | 164.9±26.8 | 163.3±26.2 | 163.7±28.1 | 164.9±25.2 | 0.775 |
| Preoperative QTc (ms) | 433.5±33.3 | 433.3±33.1 | 433.2±33.5 | 432.0±28.5 | 437.9±36.9 | 0.557 |
| Preoperative SBP (mmHg) | 129.8±16.3 | 127.8±15.3 | 130.1±16.8 a | 133.1±15.9 b | 134.7±17.3 c,† | <0.001 |
| Preoperative DBP (mmHg) | 75.5±9.8 | 75.0±10.2 | 76.2±9.5 a | 74.2±8.9 | 75.6±10.1 | 0.114 |
| Blood Loss(ml) | 700[600,1000] | 700[600,1000] | 700[600,900] | 800[600,1000] | 800[600,1000] | 0.269 |
| **Postoperative conditions** |  |  |  |  |  |  |
| CREA (μmol/L) | 77.5±16.6 | 75.5±16.8 | 77.8±16.9 a | 81.2±15.2 b | 82.9±13.8 c,† | <0.001 |
| eGFR (mL/min/1.73 m^2^) | 88.3±15.1 | 90.8±15.1 | 87.3±15.0 a | 85.4±13.8 b | 84.0±14.9 c,† | <0.001 |
| SUA (μmol/L) | 263.1±82.5 | 232.7±65.4 | 265.9±76.2 a | 304.9±86.3 b,* | 363.0±89.9 c,†,‡ | <0.001 |
| Na^+^(mmol/L) | 139.844±3.750 | 139.382±3.543 | 139.973±3.566 a | 140.316±4.512 b | 141.004±4.583 c,† | <0.001 |
| Cl^-^ (mmol/L) | 104.474±3.319 | 104.340±3.359 | 104.476±3.073 | 104.623±3.618 | 104.986±4.028 | 0.317 |
| K^+^ (mmol/L) | 4.139±0.362 | 4.141±0.340 | 4.132±0.369 | 4.183±0.391 | 4.130±0.399 | 0.664 |
| Mg^2+^(mmol/L) | 0.826±0.129 | 0.826±0.130 | 0.829±0.130 | 0.813±0.123 | 0.823±0.130 | 0.744 |
| Ca^2+^(mmol/L) | 2.004±0.141 | 2.002±0.139 | 2.007±0.141 | 2.013±0.143 | 1.988±0.149 | 0.592 |
| **In-hospital adverse outcomes** |  |  |  |  |  |  |
| Fatal arrhythmia, (%) | 1.4 | 1.2 | 1.2 | 0 | 4.7 c,†,‡ | 0.045 |
| All-cause death, (%) | 0.8 | 0.6 | 0.9 | 0 | 1.9 | 0.400 |

**Supplementary Table 6 Basic characteristics and incidence of adverse outcomes in patients with BMI<25 kg/m^2^.**

|  | **All (n=1145)** | **G1(n=495)** | **G2(n=520)** | **G3(n=75)** | **G4(n=55)** | ***P*-value** |
| --- | --- | --- | --- | --- | --- | --- |
| **General conditions** |  |  |  |  |  |  |
| Age (years) | 62.2±8.2 | 62.1±8.4 | 62.4±8.1 | 61.9±8.1 | 60.8±7.5 | 0.516 |
| Age ≥ 60 (years) , (%) | 61.0 | 60.4 | 61.7 | 62.7 | 56.4 | 0.856 |
| Male, (%) | 74.7 | 77.0 | 71.7 | 73.3 | 83.6 | 0.104 |
| BMI (kg/m^2^) | 23.0±1.6 | 22.9±1.6 | 23.0±1.5 | 23.0±1.5 | 23.3±1.4 | 0.539 |
| Smoking history, (%) | 50.8 | 50.5 | 50.4 | 48.0 | 61.8 | 0.398 |
| Drinking history, (%) | 28.5 | 28.9 | 26.9 | 37.3 | 27.3 | 0.310 |
| HTN history, (%) | 58.8 | 62.0 | 54.6 a | 64.0 | 61.8 | 0.074 |
| DM history, (%) | 36.7 | 31.9 | 39.2 a | 46.7 * | 41.8 | 0.017 |
| Insulin, (%) | 8.4 | 7.3 | 9.0 | 9.3 | 10.9 | 0.655 |
| MI history, (%) | 27.7 | 28.3 | 27.1 | 20.0 | 38.2 ‡ | 0.144 |
| PCI history, (%) | 11.0 | 11.5 | 10.8 | 9.3 | 10.9 | 0.945 |
| Stroke/TIA history, (%) | 15.1 | 14.5 | 13.5 | 25.3 b, * | 21.8 | 0.026 |
| AF history, (%) | 1.9 | 2.0 | 1.9 | 1.3 | 1.8 | 0.980 |
| COPD history, (%) | 0.3 | 0.6 | 0.2 | 0 | 0 | 0.548 |
| Euro SCORE I ≥ 6, (%) | 4.1 | 4.3 | 4.5 | 1.4 | 3.6 | 0.558 |
| **Preoperative and perioperative conditions** | | |  |  |  |  |
| TC (mmol/L) | 4.13±1.09 | 4.06±1.03 | 4.19±1.13 | 3.97±1.05 | 4.35±1.26 ‡ | 0.066 |
| TG (mmol/L) | 1.25[0.92,1.72] | 1.24[0.91,1.74] | 1.24[0.90,1.65] | 1.28[0.99,1.69] | 1.39[1.09,1.75] | 0.411 |
| HDL-C (mmol/L) | 1.05±0.26 | 1.05±0.28 | 1.05±0.26 | 1.03±0.20 | 1.02±0.25 | 0.733 |
| LDL-C (mmol/L) | 2.49±0.94 | 2.42±0.88 | 2.55±1.00 a | 2.34±0.82 | 2.73±1.07 c,‡ | 0.015 |
| CREA (μmol/L) | 72.2±13.9 | 74.5±14.2 | 70.4±13.6 a | 68.5±13.6 b | 72.5±11.9 | <0.001 |
| eGFR (mL/min/1.73 m^2^) | 91.6±12.3 | 90.0±12.6 | 92.5±12.1 a | 94.4±12.2 b | 93.4±10.2 c | 0.001 |
| eGFR ≥ 90 (mL/min/1.73 m^2^), (%) | 61.1 | 56.1 | 63.8 a | 72.0 b | 65.5 | 0.011 |
| SUA (μmol/L) | 312.8±79.2 | 352.1±73.5 | 286.7±68.8 a | 261.3±69.9 b, * | 275.4±74.6 c | <0.001 |
| HUA (%) | 10.8 | 19.8 | 4.6 a | 1.3 b | 1.8 c | <0.001 |
| Na^+^(mmol/L) | 140.357±2.423 | 140.435±2.161 | 140.279±2.664 | 140.507±2.605 | 140.193±1.988 | 0.661 |
| Cl^-^ (mmol/L) | 102.410±2.879 | 102.356±2.726 | 102.450±3.037 | 102.393±2.948 | 102.547±2.641 | 0.940 |
| K^+^ (mmol/L) | 4.136±0.363 | 4.127±0.357 | 4.153±0.366 | 4.086±0.382 | 4.134±0.370 | 0.413 |
| Mg^2+^(mmol/L) | 0.912±0.079 | 0.911±0.078 | 0.912±0.080 | 0.913±0.077 | 0.911±0.091 | 0.909 |
| Ca^2+^(mmol/L) | 2.349±0.109 | 2.355±0.109 | 2.343±0.109 | 2.354±0.109 | 2.340±0.109 | 0.336 |
| hs-CRP(mg/L) | 1.24[0.45,3.55] | 1.15[0.41,3.38] | 1.31[0.46,3.85] | 1.20[0.64,4.33] | 1.27[0.49,3.37] | 0.657 |
| LVEF (%) | 59.6±9.6 | 60.0±9.4 | 59.5±9.6 | 60.6±9.1 | 55.1±11.9 c,†,‡ | 0.003 |
| LVEF ≥ 50%, (%) | 85.6 | 85.8 | 86.6 | 89.0 | 69.1 c,†,‡ | 0.004 |
| Preoperative heart rate(bpm) | 75.7±10.1 | 75.3±10.7 | 75.8±9.5 | 77.4±10.2 | 75.4±8.5 | 0.375 |
| Preoperative PR (ms) | 162.5±25.6 | 161.5±25.5 | 162.6±24.8 | 165.9±26.3 | 166.3±32.0 | 0.342 |
| Preoperative QTc (ms) | 434.0±35.1 | 435.5±35.6 | 433.0±34.6 | 436.8±38.0 | 426.2±29.0 | 0.218 |
| Preoperative SBP (mmHg) | 127.5±15.6 | 126.7±15.4 | 127.4±15.5 | 131.0±16.3 b | 132.1±16.6 c**,**† | 0.019 |
| Preoperative DBP (mmHg) | 74.3±9.6 | 74.0±9.5 | 74.6±9.7 | 75.3±9.7 | 73.7±9.5 | 0.574 |
| Blood Loss(ml) | 700[550,960] | 700[600,900] | 600[500,900] | 800[600,1000] | 800[600,1000] | 0.119 |
| **Postoperative conditions** |  |  |  |  |  |  |
| CREA (μmol/L) | 74.9±17.1 | 73.0±18.2 | 75.2±16.1 a | 77.2±15.5 b | 85.7±13.3 c,†,‡ | <0.001 |
| eGFR (mL/min/1.73 m^2^) | 88.2±14.4 | 89.6±14.4 | 87.8±14.4 a | 86.5±14.3 | 80.9±13.1 c,†,‡ | <0.001 |
| SUA (μmol/L) | 234.4±74.9 | 211.9±64.6 | 237.1±68.4 a | 275.3±70.3 b, * | 355.4±82.9 c,†,‡ | <0.001 |
| Na^+^(mmol/L) | 139.988±4.245 | 139.790±3.999 | 140.005±4.338 | 140.723±4.966 | 140.596±4.402 | 0.220 |
| Cl^-^ (mmol/L) | 104.688±3.754 | 104.572±3.702 | 104.830±3.674 | 104.727±4.349 | 104.342±4.133 | 0.643 |
| K^+^ (mmol/L) | 4.147±0.389 | 4.156±0.389 | 4.146±0.392 | 4.146±0.406 | 4.086±0.328 | 0.665 |
| Mg^2+^(mmol/L) | 0.843±0.132 | 0.843±0.135 | 0.848±0.131 | 0.817±0.119 | 0.836±0.122 | 0.324 |
| Ca^2+^(mmol/L) | 1.980±0.156 | 1.983±0.159 | 1.978±0.155 | 1.971±0.137 | 1.991±0.175 | 0.856 |
| **In-hospital adverse outcomes** |  |  |  |  |  |  |
| Fatal arrhythmia, (%) | 1.8 | 1.2 | 1.9 | 1.3 | 7.3 c,† | 0.088 |
| All-cause death, (%) | 1.1 | 1.2 | 0.8 | 1.3 | 3.6 † | 0.442 |

**Supplementary Table 7 Basic characteristics and incidence of adverse outcomes in patients with diabetes mellitus history.**

|  | **All (n=919)** | **G1(n=329)** | **G2(n=442)** | **G3(n=71)** | **G4(n=77)** | ***P*-value** |
| --- | --- | --- | --- | --- | --- | --- |
| **General conditions** |  |  |  |  |  |  |
| Age (years) | 60.9±8.2 | 61.0±8.5 | 60.7±8.4 | 62.1±6.6 | 61.0±7.7 | 0.639 |
| Age ≥ 60 (years), (%) | 55.1 | 55.0 | 54.1 | 63.4 | 53.2 | 0.520 |
| Male, (%) | 72.5 | 76.3 | 69.7 a | 73.2 | 71.4 | 0.241 |
| BMI (kg/m^2^) | 25.6±3.0 | 25.4±3.0 | 25.5±2.9 | 25.7±3.3 | 26.6±3.2 c,† | 0.008 |
| BMI ≥ 25 (kg/m^2^), (%) | 53.8 | 51.4 | 53.3 | 50.7 | 69.7 ‡ | 0.031 |
| Smoking history, (%) | 50.3 | 52.3 | 49.8 | 49.3 | 45.5 | 0.725 |
| Drinking history, (%) | 29.5 | 34.3 | 25.3 a | 39.4 * | 23.4 ‡ | 0.007 |
| HTN history, (%) | 70.9 | 75.4 | 66.7 a | 73.2 | 74.0 | 0.059 |
| Insulin, (%) | 23.7 | 23.7 | 24.4 | 21.1 | 22.1 | 0.918 |
| MI history, (%) | 28.6 | 26.4 | 32.4 | 16.9 * | 27.3 | 0.035 |
| PCI history, (%) | 14.7 | 15.5 | 13.6 | 15.5 | 16.9 | 0.815 |
| Stroke/TIA history, (%) | 18.2 | 21.3 | 15.6 a | 23.9 | 14.3 | 0.091 |
| AF history, (%) | 2.7 | 3.3 | 1.6 | 7.0 * | 2.0 | 0.090 |
| COPD history, (%) | 0.3 | 0.3 | 0.5 | 0 | 0 | 0.761 |
| Euro SCORE I ≥ 6, (%) | 3.6 | 4.3 | 3.4 | 0 | 5.2 | 0.112 |
| **Preoperative and perioperative conditions** | | |  |  |  |  |
| TC (mmol/L) | 4.00±1.04 | 3.93±1.04 | 4.06±1.03 | 3.93±1.09 | 4.03±0.99 | 0.385 |
| TG (mmol/L) | 1.44[1.03,1.97] | 1.44[1.03,1.90] | 1.43[1.02,2.04] | 1.39[1.00,2.01] | 1.53[1.21,2.03] | 0.367 |
| HDL-C (mmol/L) | .0.98±0.24 | 0.98±0.22 | 0.98±0.26 | 0.97±0.19 | 0.95±0.23 | 0.815 |
| LDL-C (mmol/L) | 2.40±0.85 | 2.34±0.84 | 2.45±0.87 | 2.29±0.81 | 2.49±0.81 | 0.139 |
| CREA (μmol/L) | 70.7±14.3 | 74.1±14.6 | 68.7±14.1 a | 68.9±13.7 b | 68.9±11.5 c | <0.001 |
| eGFR (mL/min/1.73 m^2^) | 93.3±12.8 | 91.0±13.7 | 94.6±12.2 a | 93.6±12.4 | 95.0±11.3 c | 0.001 |
| eGFR ≥ 90 (mL/min/1.73 m^2^), (%) | 67.0 | 59.6 | 70.7 a | 70.4 | 74.0 c | 0.004 |
| SUA (μmol/L) | 312.1±85.7 | 359.6±77.5 | 292.5±75.5 a | 261.2±81.3 b,* | 269.1±86.0 c,† | <0.001 |
| HUA (%) | 12.9 | 24.6 | 6.8 a | 4.2 b | 6.5 c | <0.001 |
| Na^+^(mmol/L) | 140.329±2.436 | 140.339±2.298 | 140.313±2.459 | 140.504±2.600 | 140.213±2.743 | 0.904 |
| Cl^-^ (mmol/L) | 102.401±102.4 | 102.411±2.869 | 102.391±2.773 | 102.463±2.900 | 102.358±3.213 | 0.996 |
| K^+^ (mmol/L) | 4.115±0.368 | 4.135±0.366 | 4.123±0362 | 4.035±0.379 b | 4.064±0.390 | 0.114 |
| Mg^2+^(mmol/L) | 0.893±0.079 | 0.890±0.075 | 0.896±0.080 | 0.896±0.085 | 0.887±0.088 | 0.720 |
| Ca^2+^(mmol/L) | 2.346±0.109 | 2.350±0.114 | 2.345±0.108 | 2.331±0.102 | 2.3480.096 | 0.597 |
| hs-CRP(mg/L) | 1.43[0.57,3.85] | 1.35[0.49,3.40] | 1.60[0.57,4.00] a | 1.24[0.72,3.81] | 1.40[0.70,4.19] | 0.051 |
| LVEF (%) | 59.9±9.3 | 60.5±8.7 | 59.6±9.7 | 61.7±7.2 | 57.6±10.6 c,‡ | 0.035 |
| LVEF ≥ 50%, (%) | 87.4 | 89.3 | 86.5 | 95.7 * | 76.6 c,‡ | 0.003 |
| Preoperative heart rate(bpm) | 75.5±10.3 | 75.4±10.7 | 75.5±10.1 | 75.9±10.1 | 75.3±10.6 | 0.987 |
| Preoperative PR (ms) | 164.6±25.4 | 163.3±26.1 | 164.5±24.9 | 169.3±26.1 | 166.2±24.5 | 0.314 |
| Preoperative QTc (ms) | 435.2±34.7 | 436.0±36.4 | 434.3±34.4 | 432.9±28.8 | 438.8±39.9 | 0.651 |
| Preoperative SBP (mmHg) | 128.8±16.4 | 127.1±14.5 | 128.3±17.3 | 132.4±16.9 b,* | 135.5±15.9 c,† | <0.001 |
| Preoperative DBP (mmHg) | 74.3±9.6 | 73.9±9.6 | 74.3±9.6 | 75.4±9.7 | 75.3±9.2 | 0.506 |
| Blood Loss(ml) | 700[600,900] | 700[600,900] | 700[550,800] | 800[600,1000] | 800[600,1000] | 0.315 |
| **Postoperative conditions** |  |  |  |  |  |  |
| CREA (μmol/L) | 75.4±17.4 | 74.3±18.3 | 74.5±17.0 | 78.2±17.4 | 82.2±13.2 c,† | 0.001 |
| eGFR (mL/min/1.73 m^2^) | 88.4±15.3 | 89.6±15.5 | 89.1±15.1 | 85.6±15.2 b | 82.0±14.5 c,† | <0.001 |
| SUA (μmol/L) | 246.3±79.8 | 220.7±63.6 | 244.0±74.0 a | 272.9±81.0 b.* | 344.7±91.3 c,†,‡ | <0.001 |
| Na^+^(mmol/L) | 139.741±3.822 | 139.468±3.679 | 139.779±3.820 | 139.945±4.299 | 140.484±3.914 c | 0.186 |
| Cl^-^ (mmol/L) | 104.558±3.428 | 104.441±3.524 | 104.652±3.290 | 104.378±3.767 | 104.686±3.499 | 0.799 |
| K^+^ (mmol/L) | 4.130±0.365 | 4.130±0.325 | 4.122±0.381 | 4.167±0.430 | 4.141±0.374 | 0.805 |
| Mg^2+^(mmol/L) | 0.831±0.128 | 0.828±0.127 | 0.835±0.130 | 0.829±0.119 | 0.820±0.125 | 0.720 |
| Ca^2+^(mmol/L) | 1.986±0.146 | 1.984±0.145 | 1.984±0.146 | 1.983±0.134 | 2.003±0.164 | 0.779 |
| **In-hospital adverse outcomes** |  |  |  |  |  |  |
| Fatal arrhythmia, (%) | 1.4 | 0.6 | 1.6 | 0 | 5.2 c,† | 0.033 |
| All-cause death, (%) | 0.9 | 0.6 | 0.9 | 0 | 2.6 | 0.351 |

**Supplementary Table 8 Basic characteristics and incidence of adverse outcomes in patients without diabetes mellitus history.**

|  | **All (n=1534)** | **G1(n=701)** | **G2(n=652)** | **G3(n=92)** | **G4(n=89)** | ***P*-value** |
| --- | --- | --- | --- | --- | --- | --- |
| **General conditions** |  |  |  |  |  |  |
| Age (years) | 60.9±8.9 | 60.5±9.0 | 61.8±8.6 a | 59.5±9.3 * | 58.2±9.0 c,† | <0.001 |
| Age ≥ 60 (years), (%) | 54.2 | 52.2 | 58.7 a | 46.7 * | 44.9 † | 0.009 |
| Male, n (%) | 79.3 | 81.5 | 76.1 a | 79.3 | 85.4 † | 0.044 |
| BMI (kg/m2) | 25.4±3.1 | 25.3±3.0 | 25.4±3.1 | 25.5±3.0 | 26.2±3.4 c,† | 0.068 |
| BMI ≥ 25 (kg/m^2^), (%) | 52.0 | 51.1 | 51.0 | 56.0 | 62.4 c,† | 0.193 |
| Smoking history, (%) | 52.7 | 51.1 | 52.6 | 57.6 | 60.7 | 0.272 |
| Drinking history, (%) | 28.2 | 29.5 | 26.1 | 32.6 | 29.2 | 0.393 |
| HTN history, (%) | 61.7 | 63.5 | 60.9 | 60.9 | 53.9 | 0.331 |
| MI history, (%) | 27.9 | 28.1 | 25.5 | 32.6 | 39.3 c,† | 0.033 |
| PCI history, (%) | 11.6 | 13.4 | 10.3 | 9.8 | 9.0 | 0.237 |
| Stroke/TIA history, (%) | 13.3 | 11.6 | 15.0 | 14.1 | 13.5 | 0.308 |
| AF history, (%) | 2.2 | 2.0 | 2.8 | 0 | 2.2 | 0.164 |
| COPD history, (%) | 0.4 | 0.3 | 0.2 | 1.1 | 2.2 | 0.114 |
| Euro SCORE I ≥ 6, (%) | 3.3 | 3.2 | 3.7 | 2.2 | 2.2 | 0.779 |
| **Preoperative and perioperative conditions** | | |  |  |  |  |
| TC (mmol/L) | 4.24±1.14 | 4.18±1.13 | 4.26±1.14 | 4.26±1.00 | 4.51±1.23 c,† | 0.066 |
| TG (mmol/L) | 1.34[1.01,1.94] | 1.35[1.01,1.96] | 1.31[0.97,1.88] | 1.43[1.10,2.08] * | 1.50[1.14,2.23] ‡ | 0.018 |
| HDL-C (mmol/L) | 1.02±0.24 | 1.02±0.26 | 1.04±0.23 | 1.01±0.20 | 0.99±0.22 | 0.155 |
| LDL-C (mmol/L) | 2.59±0.98 | 2.53±0.96 | 2.62±1.02 | 2.56±0.78 | 2.85±1.01 c,†,‡ | 0.028 |
| CREA (μmol/L) | 74.4±13.6 | 76.1±13.7 | 72.9±13.5 a | 72.5±12.3 b | 74.1±13.7 | <0.001 |
| eGFR (mL/min/1.73 m^2^) | 91.2±12.3 | 90.3±12.7 | 91.5±12.1 a | 93.5±11.3 b | 94.1±11.5 c | 0.007 |
| eGFR ≥ 90 (mL/min/1.73 m^2^), (%) | 59.8 | 56.3 | 61.0 | 69.6 b | 67.4 c | 0.020 |
| SUA (μmol/L) | 332.5±80.7 | 366.1±76.8 | 307.4±71.9 a | 292.1±77.6 b | 293.7±72.6 c | <0.001 |
| HUA (%) | 15.5 | 24.5 | 8.3 a | 7.6 b | 5.6 c | <0.001 |
| Na^+^(mmol/L) | 140.402±2.476 | 140.417±2.226 | 140.341±2.729 | 140.711±2.468 | 140.412±2.415 | 0.604 |
| Cl^-^ (mmol/L) | 102.603±2.912 | 102.623±2.745 | 102.562±3.040 | 102.642±2.810 | 102.715±3.360 | 0.958 |
| K^+^ (mmol/L) | 4.130±0.347 | 4.103±0.336 | 4.155±0.356 a | 4.089±0.351 | 4.195±0.343 b,‡ | 0.007 |
| Mg^2+^(mmol/L) | 0.909±0.078 | 0.910±0.079 | 0.910±0.078 | 0.897±0.068 | 0.905±0.080 | 0.444 |
| Ca^2+^(mmol/L) | 2.345±0.109 | 2.348±0.110 | 2.341±0.109 | 2.356±0.095 | 2.334±0.118 | 0.333 |
| hs-CRP(mg/L) | 1.59[0.64,4.25] | 1.52[0.64,4.09] | 1.56[0.62,4.15] | 2.00[0.76,7.53] | 2.11[0.81,5.66] c,† | 0.028 |
| LVEF (%) | 60.4±9.2 | 60.8±9.0 | 60.7±8.9 | 60.2±9.6 | 55.5±11.9 c,†,‡ | <0.001 |
| LVEF ≥ 50%, (%) | 88.5 | 88.7 | 91.4 | 85.7 | 69.7 c,†,‡ | <0.001 |
| Preoperative heart rate(bpm) | 75.4±10.2 | 75.2±10.5 | 75.7±10.1 | 75.4±9.6 | 75.0±9.1 | 0.732 |
| Preoperative PR (ms) | 162.4±26.3 | 163.0±26.1 | 161.8±25.8 | 160.9±27.5 | 164.8±30.5 | 0.642 |
| Preoperative QTc (ms) | 433.0±34.1 | 433.8±33.9 | 432.4±33.9 | 434.9±36.2 | 429.0±34.7 | 0.562 |
| Preoperative SBP (mmHg) | 128.7±15.9 | 127.4±15.7 | 129.2±15.8 a | 132.0±15.4 b | 132.4±17.8 c | 0.003 |
| Preoperative DBP (mmHg) | 75.4±9.8 | 74.9±10.0 | 76.2±9.7 a | 74.3±9.1 | 74.6±10.4 | 0.048 |
| Blood Loss(ml) | 700[600,1000] | 700[600,980] | 700[600,1000] | 800[600,1000] | 800[600,1000] | 0.336 |
| **Postoperative conditions** |  |  |  |  |  |  |
| CREA (μmol/L) | 76.9±16.6 | 74.5±17.2 | 78.0±16.2 a | 80.3±13.7 b | 85.2±14.1 c,†,‡ | <0.001 |
| eGFR (mL/min/1.73 m^2^) | 88.0±14.5 | 90.4±14.4 | 86.5±14.4 a | 86.2±13.0 b | 83.3±14.1 c | <0.001 |
| SUA (μmol/L) | 251.7±80.4 | 223.9±66.7 | 257.3±73.4 a | 306.0±77.2 b,* | 373.6±82.3 c,†,‡ | <0.001 |
| Na^+^(mmol/L) | 140.025±4.075 | 139.639±3.815 | 140.142±4.031 a | 140.948±4.972 b | 141.216±4.886 c,† | <0.001 |
| Cl^-^ (mmol/L) | 104.585±3.585 | 104.448±3.523 | 104.658±3.440 | 104.891±4.088 | 104.806±4.460 | 0.519 |
| K^+^ (mmol/L) | 4.150±0.380 | 4.157±0.383 | 4.150±0.378 | 4.162±0.372 | 4.094±0.378 | 0.533 |
| Mg^2+^(mmol/L) | 0.836±0.133 | 0.838±0.135 | 0.840±0.132 | 0.804±0.121 b,* | 0.833±0.128 | 0.112 |
| Ca^2+^(mmol/L) | 1.996±0.151 | 1.996±0.152 | 1.998±0.151 | 2.003±0.147 | 1.976±0.148 | 0.613 |
| **In-hospital adverse outcomes** |  |  |  |  |  |  |
| Fatal arrhythmia, (%) | 1.8 | 1.6 | 1.5 | 1.1 | 5.6 c,† | 0.138 |
| All-cause death, (%) | 1.0 | 1.1 | 0.8 | 1.1 | 2.2 | 0.674 |

**Supplementary Table 9 Basic characteristics and incidence of adverse outcomes in patients with hypertension history.**

|  | **All (n=1598)** | **G1(n=693)** | **G2(n=692)** | **G3(n=108)** | **G4(n=105)** | ***P*-value** |
| --- | --- | --- | --- | --- | --- | --- |
| **General conditions** |  |  |  |  |  |  |
| Age (years) | 61.6±8.4 | 61.3±8.7 | 62.2±8.3 | 61.2±7.4 | 60.5±8.2 | 0.105 |
| Age ≥ 60 (years), (%) | 57.7 | 55.7 | 60.8 | 54.6 | 53.3 | 0.160 |
| Sex (Male), (%) | 74.2 | 77.8 | 70.2 a | 75.0 | 75.2 | 0.015 |
| BMI (kg/m^2^) | 25.8±3.1 | 25.6±3.0 | 25.8±3.1 | 25.6±3.2 | 26.7±3.2 c,†,‡ | 0.003 |
| BMI ≥ 25 (kg/m^2^), (%) | 57.2 | 54.9 | 58.4 | 55.1 | 66.7 c | 0.123 |
| Smoking history, (%) | 50.8 | 52.1 | 48.6 | 56.5 | 51.4 | 0.352 |
| Drinking history, (%) | 28.8 | 32.2 | 24.7 a | 37.0 * | 24.8 | 0.003 |
| DM history, (%) | 40.8 | 35.8 | 42.6 a | 48.1 b | 54.3 c,† | <0.001 |
| Insulin, (%) | 8.7 | 8.1 | 9.0 | 9.3 | 10.5 | 0.837 |
| MI history, (%) | 26.1 | 25.7 | 26.0 | 25.0 | 30.5 | 0.759 |
| PCI history, (%) | 12.2 | 13.1 | 10.8 | 14.8 | 12.4 | 0.484 |
| Stroke/TIA history, (%) | 17.5 | 17.3 | 17.5 | 17.6 | 19.0 | 0.979 |
| AF history, (%) | 2.6 | 2.7 | 2.3 | 3.7 | 1.9 | 0.800 |
| COPD history, (%) | 0.3 | 0.3 | 0.3 | 0.9 | 0 | 0.674 |
| Euro SCORE I ≥ 6, (%) | 4.0 | 4.8 | 3.6 | 0.9 | 4.8 | 0.165 |
| **Preoperative and perioperative conditions** | | |  |  |  |  |
| TC (mmol/L) | 4.12±1.08 | 4.09±1.10 | 4.16±1.05 | 4.09±1.08 | 4.19±1.12 | 0.545 |
| TG (mmol/L) | 1.40 [1.04,2.00] | 1.43 [1.03,1.98] | 1.37 [1.04,1.99] | 1.42 [1.01,2.00] | 1.54[1.14,2.28] | 0.658 |
| HDL-C (mmol/L) | 1.00±0.24 | 1.00±0.26 | 1.00±0.23 | 1.01±0.20 | 0.96±0.22 | 0.361 |
| LDL-C (mmol/L) | 2.49±0.90 | 2.46±0.90 | 2.52±0.89 | 2.44±0.85 | 2.61±0.92 | 0.273 |
| CREA (μmol/L) | 73.3±14.3 | 75.9±14.2 | 71.3±14.2 a | 72.0±13.4 b | 70.5±13.2 c | <0.001 |
| eGFR (mL/min/1.73 m^2^) | 90.9±12.5 | 89.5±13.1 | 91.7±12.0 a | 92.0±11.3 b | 94.6±11.0 c,† | <0.001 |
| eGFR ≥ 90 (mL/min/1.73 m^2^), (%) | 59.6 | 54.3 | 62.2 a | 65.7 b | 70.5 c | <0.001 |
| SUA (μmol/L) | 326.3±84.0 | 367.3±75.0 | 300.0±74.9 a | 280.6±82.2 b,* | 257.7±78.5 c,† | <0.001 |
| H-SUA, (%) | 15.3 | 25.5 | 8.1 a | 7.4 b | 3.8 c | <0.001 |
| Na^+^(mmol/L) | 140.416±2.521 | 140.401±2.307 | 140.331±2.721 | 140.935±2.471 b,* | 140.545±2.515 | 0.128 |
| Cl^-^ (mmol/L) | 102.563±2.913 | 102.560±2.754 | 102.472±3.045 | 102.853±2.769 | 102.891±3.182 | 0.379 |
| K^+^ (mmol/L) | 4.122±0.357 | 4.121±0.345 | 4.131±0.365 | 4.077±0.363 | 4.108±0.375 | 0.508 |
| Mg^2+^(mmol/L) | 0.902±0.079 | 0.907±0.079 | 0.900±0.078 | 0.899±0.074 | 0.889±0.086 c | 0.114 |
| Ca^2+^(mmol/L) | 2.346±0.113 | 2.349±0.115 | 2.345±0.113 | 2.347±0.102 | 2.337±0.114 | 0.727 |
| hs-CRP(mg/L) | 1.62[0.65,4.18] | 1.53[0.63,3.98] | 1.74[0.66,4.27] | 1.55[0.72,4.30] | 1.54[0.64,4.56] | 0.394 |
| LVEF (%) | 61.1±8.7 | 61.4±8.5 | 61.2±8.5 | 61.7±8.5 | 57.6±10.5 c,†,‡ | <0.001 |
| LVEF ≥ 50%, (%) | 90.3 | 90.9 | 92.0 | 90.6 | 75.2 c,†,‡ | <0.001 |
| Preoperative heart rate(bpm) | 75.2±10.3 | 75.0±10.2 | 75.4±10.3 | 75.5±10.2 | 75.2±10.3 | 0.848 |
| Preoperative PR (ms) | 163.8±26.6 | 163.9±27.5 | 162.8±25.1 | 167.1±29.6 | 166.2±27.1 | 0.329 |
| Preoperative QTc (ms) | 433.2±34.1 | 433.6±35.1 | 433.1±34.0 | 428.5±26.5 | 436.6±34.7 | 0.360 |
| Preoperative SBP (mmHg) | 130.3±16.3 | 128.6±15.7 | 130.5±16.6 a | 133.7±16.6 b | 136.3±15.9 c,† | <0.001 |
| Preoperative DBP (mmHg) | 75.4±10.0 | 74.9±10.2 | 75.8±10.0 | 75.1±9.3 | 76.1±10.0 | 0.315 |
| Blood Loss(ml) | 700[600,900] | 700[600,900] | 700[563,900] | 800[600,975] | 800[600,1000] | 0.556 |
| **Postoperative conditions** |  |  |  |  |  |  |
| CREA (μmol/L) | 76.8±17.3 | 74.9±18.0 | 77.1±17.0 a | 79.6±15.6 b | 83.2±13.6 c,† | <0.001 |
| eGFR (mL/min/1.73 m^2^) | 86.9±14.6 | 88.9±14.8 | 86.0±14.5 a | 85.2±13.3 b | 82.0±13.1 c,† | <0.001 |
| SUA (μmol/L) | 248.7±79.4 | 224.8±64.9 | 249.9±75.2 a | 293.2±81.6 b,* | 353.4±86.0 c,†,‡ | <0.001 |
| Na^+^(mmol/L) | 139.921±4.004 | 139.821±3.831 | 139.833±3.974 | 140.814±4.774 b,* | 140.238±4.367 | 0.080 |
| Cl^-^ (mmol/L) | 104.581±3.516 | 104.581±3.483 | 104.619±3.367 | 104.505±4.055 | 104.403±4.093 | 0.940 |
| K^+^ (mmol/L) | 4.148±0.380 | 4.152±0.378 | 4.145±0.381 | 4.155±0.388 | 4.132±0.378 | 0.951 |
| Mg^2+^(mmol/L) | 0.836±0.132 | 0.839±0.136 | 0.838±0.133 | 0.818±0.112 | 0.815±0.122 | 0.197 |
| Ca^2+^(mmol/L) | 1.995±0.152 | 1.997±0.152 | 1.992±0.151 | 1.991±0.150 | 2.000±0.164 | 0.911 |
| **In-hospital adverse outcomes** |  |  |  |  |  |  |
| Fatal arrhythmia, (%) | 1.5 | 1.4 | 1.3 | 0.9 | 3.8 | 0.369 |
| All-cause death, (%) | 1.1 | 1.0 | 0.9 | 0.9 | 2.9 | 0.474 |

**Supplementary Table 10 Basic characteristics and incidence of adverse outcomes in patients without hypertension history.**

|  | **All (n=855)** | **G1(n=337)** | **G2(n=402)** | **G3(n=55)** | **G4(n=61)** | ***P*-value** |
| --- | --- | --- | --- | --- | --- | --- |
| **General conditions** |  |  |  |  |  |  |
| Age (years) | 59.5±8.9 | 59.4±8.9 | 59.9±8.7 | 59.5±9.9 | 57.8±8.9 | 0.381 |
| Age ≥ 60 (years), (%) | 48.7 | 47.8 | 50.0 | 52.7 | 41.0 | 0.532 |
| Sex (Male), (%) | 81.5 | 84.0 | 79.1 | 80.0 | 85.2 | 0.314 |
| BMI (kg/m^2^) | 24.9±2.9 | 24.8±2.9 | 24.8±2.9 | 25.5±3.1 | 25.9±3.5 c,† | 0.018 |
| BMI ≥ 25 (kg/m^2^), (%) | 44.2 | 43.5 | 40.9 | 50.9 | 64.4 c,† | 0.005 |
| Smoking history, (%) | 53.6 | 50.1 | 56.5 | 49.1 | 57.4 | 0.291 |
| Drinking history, (%) | 28.5 | 28.8 | 27.6 | 32.7 | 29.5 | 0.878 |
| DM history, (%) | 31.2 | 24.0 | 36.6 a | 34.5 | 32.8 | 0.003 |
| Insulin, (%) | 9.2 | 6.5 | 11.4 a | 9.1 | 9.8 | 0.151 |
| MI history, (%) | 32.0 | 31.5 | 32.1 | 27.3 | 39.3 | 0.547 |
| PCI history, (%) | 13.8 | 16.0 | 12.9 | 7.3 | 13.1 | 0.302 |
| Stroke/TIA history, (%) | 10.6 | 9.2 | 11.4 | 20 c | 4.9 ‡ | 0.043 |
| AF history, (%) | 2.1 | 1.8 | 2.2 | 1.8 | 3.3 | 0.899 |
| COPD history, (%) | 0.5 | 0.3 | 0.2 | 0 | 3.3 c,† | 0.129 |
| Euro SCORE I ≥ 6, (%) | 2.2 | 0.9 | 3.5 a | 1.9 | 1.6 | 0.102 |
| **Preoperative and perioperative conditions** | | |  |  |  |  |
| TC (mmol/L) | 4.20±1.16 | 4.14±1.13 | 4.21±1.20 | 4.16±1.00 | 4.45±1.19 c | 0.264 |
| TG (mmol/L) | 1.32[0.98,1.88] | 1.29[0.99,1.82] | 1.30[0.91,1.88] | 1.39[1.13,2.25] b,* | 1.50[1.20,2.01] | 0.017 |
| HDL-C (mmol/L) | 1.01±0.25 | 1.01±0.23 | 1.03±0.27 | 0.96±0.19 * | 0.99±0.22 | 0.167 |
| LDL-C (mmol/L) | 2.57±1.01 | 2.50±0.98 | 2.60±1.07 | 2.45±0.72 | 2.81±0.96 c | 0.104 |
| CREA (μmol/L) | 72.5±13.4 | 74.7±13.4 | 71.1±13.3 a | 68.8±12.1 b | 73.7±12.4 ‡ | <0.001 |
| eGFR (mL/min/1.73 m^2^) | 93.9±12.5 | 92.7±12.7 | 94.5±12.3 | 96.6±12.1 b | 94.5±12.2 | 0.083 |
| eGFR ≥ 90 (mL/min/1.73 m^2^), (%) | 67.8 | 63.5 | 69.7 | 78.2 b | 70.5 | 0.093 |
| SUA (μmol/L) | 322.2±81.6 | 357.2±80.8 | 303.8±71.5 a | 274.9±77.4 b,* | 293.5±81.3 c | <0.001 |
| H-SUA, (%) | 13.1 | 22.6 | 7.0 | 3.6 | 9.8 c,†,‡ | <0.001 |
| Na^+^(mmol/L) | 140.298±2.344 | 140.375±2.127 | 140.329±2.455 | 140.004±2.525 | 140.933±2.627 | 0.428 |
| Cl^-^ (mmol/L) | 102.461±2.868 | 102.545±2.759 | 102.529±2.857 | 101.998±2.937 | 101.961±3.407 | 0.283 |
| K^+^ (mmol/L) | 4.129±0.351 | 4.097±0.348 | 4.161±0.347 a | 4.044±0.366 * | 4.179±0.360 ‡ | 0.014 |
| Mg^2+^(mmol/L) | 0.905±0.079 | 0.898±0.077 | 0.911±0.080 a | 0.892±0.080 | 0.911±0.081 | 0.088 |
| Ca^2+^(mmol/L) | 2.343±0.101 | 2.348±0.104 | 2.339±0.101 | 2.341±0.092 | 2.348±0.098 | 0.645 |
| hs-CRP(mg/L) | 1.35[0.55,3.76] | 1.17[0.51,3.64] | 1.34[0.52,3.61] | 1.32[0.77,5.64] | 1.99[0.86,4.29] | 0.174 |
| LVEF (%) | 58.7±10.0 | 59.3±9.5 | 58.7±10.1 | 59.2±8.7 | 54.7±12.4 c,†,‡ | 0.010 |
| LVEF ≥ 50%, (%) | 84.0 | 84.8 | 85.0 | 88.9 | 68.9 c,†,‡ | 0.008 |
| Preoperative heart rate(bpm) | 75.8±10.2 | 75.8±11.1 | 76.0±9.7 | 75.9±9.0 | 75.0±9.1 | 0.911 |
| Preoperative PR (ms) | 162.3±24.8 | 161.4±23.0 | 163.1±26.1 | 159.3±20.8 | 164.3±29.1 | 0.598 |
| Preoperative QTc (ms) | 434.9±34.8 | 436.4±34.0 | 433.2±34.3 | 445.2±41.5 * | 428.4±34.0 ‡ | 0.038 |
| Preoperative SBP (mmHg) | 125.9±15.3 | 124.6±14.4 | 125.9±15.6 | 129.2±14.7 b | 129.5±17.9 c | 0.039 |
| Preoperative DBP (mmHg) | 74.2±9.2 | 73.8±9.2 | 74.8±9.0 | 74.2±9.6 | 72.8±9.3 | 0.322 |
| Blood Loss(ml) | 800[600,1000] | 800[600,1000] | 700[588,1000] | 800[600,1000] | 800[600,1000] | 0.155 |
| **Postoperative conditions** |  |  |  |  |  |  |
| CREA (μmol/L) | 75.6±16.2 | 73.3±16.5 | 75.7±15.8 a | 79.0±15.1 b | 84.8±14.0 c,† | <0.001 |
| eGFR (mL/min/1.73 m^2^) | 90.5±14.9 | 92.7±14.4 | 90.2±14.7 a | 87.4±15.2 b | 84.0±16.1 c,† | <0.001 |
| SUA (μmol/L) | 251.5±81.8 | 219.0±67.2 | 255.5±71.6 a | 288.3±78.4 b,* | 372.0±89.6 c,†,‡ | <0.001 |
| Na^+^(mmol/L) | 139.913±3.946 | 139.097±3.602 | 140.277±3.897 a | 139.915±4.540 | 141.97±4.449 c,†,‡ | <0.001 |
| Cl^-^ (mmol/L) | 104.565±3.547 | 104.567±3.589 | 104.719±3.403 a | 104.980±3.743 | 105.348±3.883 c | 0.034 |
| K^+^ (mmol/L) | 4.133±0.365 | 4.138±0.340 | 4.128±0.377 | 4.182±0.416 | 4.088±0.374 | 0.555 |
| Mg^2+^(mmol/L) | 0.832±0.128 | 0.825±0.126 | 0.839±0.127 | 0.807±0.136 | 0.846±0.133 | 0.180 |
| Ca^2+^(mmol/L) | 1.988±0.144 | 1.982±0.146 | 1.994±0.146 | 2.002±0.124 | 1.969±0.142 | 0.470 |
| **In-hospital adverse outcomes** |  |  |  |  |  |  |
| Fatal arrhythmia, (%) | 1.9 | 0.9 | 2.0 | 0 | 8.2 c,†,‡ | 0.009 |
| All-cause death, (%) | 0.8 | 0.9 | 0.7 | 0.0 | 1.6 | 0.719 |

**Supplementary Table 11 Basic characteristics and incidence of adverse outcomes in patients with myocardial infarction history.**

|  | **All (n=691)** | **G1(n=284)** | **G2(n=309)** | **G3(n=42)** | **G4(n=56)** | ***P*-value** |
| --- | --- | --- | --- | --- | --- | --- |
| **General conditions** |  |  |  |  |  |  |
| Age (years) | 59.3±9.1 | 60.0±8.9 | 59.4±8.9 | 58.1±8.7 | 57.5±10.1 | 0.312 |
| Age ≥ 60 (years), (%) | 47.0 | 47.5 | 48.2 | 42.9 | 41.1 | 0.730 |
| Male, (%) | 82.2 | 83.1 | 81.6 | 78.6 | 83.9 | 0.864 |
| BMI (kg/m2) | 25.5±3.0 | 25.1±2.9 | 25.5±2.9 | 26.7±3.2 b,* | 26.0±2.9 c | 0.006 |
| BMI ≥ 25 (kg/m^2^), (%) | 53.5 | 49.6 | 54.2 | 64.3 | 61.1 | 0.175 |
| Smoking history, (%) | 60.1 | 59.2 | 60.8 | 50.0 | 67.9 | 0.338 |
| Drinking history, (%) | 28.2 | 29.9 | 27.8 | 28.6 | 21.4 | 0.635 |
| HTN history, (%) | 60.3 | 62.7 | 58.3 | 64.3 | 57.1 | 0.632 |
| DM, (%) | 38.1 | 30.6 | 46.3 a | 28.6 * | 37.5 | 0.001 |
| Insulin, (%) | 9.4 | 8.5 | 10.4 | 9.5 | 8.9 | 0.885 |
| PCI history,(%) | 19.2 | 21.1 | 18.1 | 19.0 | 16.1 | 0.738 |
| Stroke/TIA history, (%) | 15.3 | 17.3 | 14.6 | 9.5 | 14.3 | 0.555 |
| AF history, (%) | 2.0 | 2.1 | 1.6 | 2.4 | 3.6 | 0.834 |
| COPD history, (%) | 0.4 | 0.4 | 0.6 | 0 | 0 | 0.757 |
| Euro SCORE I ≥ 6, (%) | 3.6 | 3.2 | 4.2 | 0 | 5.4 | 0.263 |
| **Preoperative and perioperative conditions** | | |  |  |  |  |
| TC (mmol/L) | 4.09±1.05 | 4.05±1.06 | 4.10±1.06 | 3.95±0.83 | 4.32±1.05 | 0.283 |
| TG (mmol/L) | 1.44[1.04,2.01] | 1.47[1.05,2.02] | 1.39[1.01,1.94] | 1.63[1.08,2.18] | 1.43[1.08,2.22] | 0.618 |
| HDL-C (mmol/L) | 0.97±0.23 | 0.98±0.22 | 0.97±0.24 | 0.93±0.20 | 0.95±0.22 | 0.358 |
| LDL-C (mmol/L) | 2.50±0.90 | 2.43±0.87 | 2.55±0.94 | 2.37±0.70 | 2.74±0.87 c ,‡ | 0.067 |
| CREA (μmol/L) | 75.2±14.4 | 77.0±14.6 | 73.7±13.9 a | 74.7±13.2 | 74.4±15.3 | 0.041 |
| eGFR (mL/min/1.73 m^2^) | 92.0±13.0 | 90.3±13.1 | 93.0±12.5 a | 92.7±14.2 | 94.0±13.6 | 0.050 |
| eGFR ≥ 90 (mL/min/1.73 m^2^), (%) | 61.4 | 56.3 | 64.7 a | 66.7 | 64.3 | 0.158 |
| SUA (μmol/L) | 330.6±84.8 | 369.3±76.8 | 330.1±77.2 a | 290.9±84.8 b | 277.0±83.0 c,† | <0.001 |
| HUA, (%) | 15.8 | 25.7 | 9.7 a | 11.9 | 1.8 c,‡ | <0.001 |
| Na^+^(mmol/L) | 140.314±2.365 | 140.257±2.359 | 140.399±2.491 | 140.276±2.348 | 140.157±1.622 | 0.844 |
| Cl^-^ (mmol/L) | 102.400±2.805 | 102.425±2.636 | 102.362±3.031 | 102.336±2.987 | 102.530±2.187 | 0.974 |
| K^+^ (mmol/L) | 4.146±0.363 | 4.147±0.354 | 4.146±0.371 | 4.124±0.404 | 4.154±0.340 | 0.980 |
| Mg^2+^(mmol/L) | 0.900±0.081 | 0.899±0.082 | 0.900±0.079 | 0.894±0.075 | 0.914±0.088 | 0.610 |
| Ca^2+^(mmol/L) | 2.346±0.110 | 2.340±0.111 | 2.345±0.109 | 2.366±0.107 | 2.364±0.114 | 0.302 |
| hs-CRP(mg/L) | 1.69[0.65.4.64] | 1.37[0.55,4.23] | 1.78[0.73,4.80] a | 1.77[0.75,5.92] | 2.54[1.13,10.58] c | 0.009 |
| LVEF (%) | 55.8±10.5 | 56.7±10.3 | 55.9±10.4 | 57.1±9.2 | 49.8±11.1 c,†,‡ | <0.001 |
| LVEF ≥ 50%,(%) | 75.3 | 77.7 | 76.9 | 78.6 | 51.8 c,†,‡ | <0.001 |
| Preoperative heart rate(bpm) | 75.2±10.2 | 74.9±10.2 | 75.6±10.2 | 74.9±10.3 | 75.5±9.4 | 0.860 |
| Preoperative PR (ms) | 163.3±24.9 | 163.8±24.6 | 162.6±23.8 | 163.6±30.8 | 164.4±27.2 | 0.927 |
| Preoperative QTc (ms) | 436.6±35.6 | 435.6±35.7 | 438.0±36.2 | 434.4±39.3 | 435.5±34.7 | 0.810 |
| Preoperative SBP (mmHg) | 127.0±16.2 | 126.5±16.6 | 126.2±15.8 | 129.4±13.9 | 131.9±17.7 c,† | 0.070 |
| Preoperative DBP (mmHg) | 74.2±9.5 | 74.3±9.8 | 74.2±9.5 | 73.8±7.7 | 73.8±10.0 | 0.969 |
| Blood Loss(ml) | 700[600,900] | 700[563,900] | 700[500,900] | 800[600,1000] | 800[600,1000] | 0.393 |
| **Postoperative conditions** |  |  |  |  |  |  |
| CREA (μmol/L) | 78.1±17.1 | 75.4±17.6 | 78.7±16.4 a | 81.0±17.9 b | 85.9±15.4 c,† | <0.001 |
| eGFR (mL/min/1.73 m^2^) | 88.6±15.2 | 90.5±14.4 | 88.1±15.3 a | 87.1±17.0 | 82.8±16.1 c,† | 0.004 |
| SUA (μmol/L) | 257.3±83.2 | 226.5±63.5 | 260.5±76.8 a | 302.8±84.4 b,* | 362.4±100.0 c,†,‡ | <0.001 |
| Na^+^(mmol/L) | 140.018±4.307 | 139.544±4.070 | 140.145±4.365 a | 140.274±4.920 | 141.505±4.372 c,† | 0.015 |
| Cl^-^ (mmol/L) | 104.505±3.555 | 104.226±3.445 | 104.609±3.452 | 104.352±3.881 | 105.446±4.247 c | 0.112 |
| K^+^ (mmol/L) | 4.149±0.379 | 4.176±0.381 | 4.117±0.371 | 4.183±0.458 | 4.159±0.336 | 0.276 |
| Mg^2+^(mmol/L) | 0.836±0.132 | 0.831±0.128 | 0.847±0.135 | 0.800±0.127 * | 0.824±0.140 | 0.115 |
| Ca^2+^(mmol/L) | 1.992±0.147 | 1.998±0.138 | 1.991±0.152 | 1.983±0.144 | 1.974±0.163 | 0.710 |
| **In-hospital adverse outcomes** |  |  |  |  |  |  |
| Fatal arrhythmia, (%) | 2.6 | 1.8 | 2.9 | 0 | 7.1 c | 0.096 |
| All-cause death, (%) | 1.6 | 0.7 | 1.9 | 0 | 5.4 c | 0.089 |

**Supplementary Table 12 Basic characteristics and incidence of adverse outcomes in patients without myocardial infarction history.**

|  | **All (n=1762)** | **G1(n=746)** | **G2(n=785)** | **G3(n=121)** | **G4(n=110)** | ***P*-value** |
| --- | --- | --- | --- | --- | --- | --- |
| **General conditions** |  |  |  |  |  |  |
| Age (years) | 61.5±8,4 | 61.1±8,8 | 62.1±8.2 a | 61.5±7.6 | 60.5±7.4 | 0.054 |
| Age ≥ 60 (years), (%) | 57.5 | 55.2 | 60.3 a | 57.9 | 52.7 | 0.169 |
| Male, (%) | 74.6 | 78.6 | 70.3 a | 76.0 | 76.4 | 0.003 |
| BMI (kg/m^2^) | 25.5±3.1 | 25.4±3.0 | 25.4±3.1 | 25.2±3.0 | 26.6±3.5 c,†,‡ | 0.001 |
| BMI ≥ 25 (kg/m^2^), (%) | 52.3 | 51.8 | 51.0 | 50.0 | 68.2 c,†,‡ | 0.008 |
| Smoking history, (%) | 48.5 | 48.5 | 47.8 | 55.4 | 46.4 | 0.448 |
| Drinking history, (%) | 28.9 | 31.5 | 25.0 a | 38.0 b | 28.1 | 0.004 |
| HTN history, (%) | 67.0 | 69.0 | 65.2 | 66.9 | 66.4 | 0.468 |
| DM history, (%) | 37.2 | 32.4 | 38.1 a | 48.8 b,* | 50.9 c,† | <0.001 |
| Insulin, (%) | 8.7 | 7.2 | 9.7 | 9.1 | 10.9 | 0.300 |
| PCI history, (%) | 10.2 | 11.4 | 9.0 | 9.9 | 10.9 | 0.499 |
| Stroke/TIA history, (%) | 15.0 | 13.7 | 15.5 | 21.5 b | 13.6 | 0.148 |
| AF history, (%) | 2.6 | 2.5 | 2.5 | 3.3 | 1.8 | 0.915 |
| COPD history, (%) | 0.3 | 0.3 | 0.1 | 0.8 | 1.8 c,† | 0.134 |
| Euro SCORE I≧6, (%) | 3.3 | 3.6 | 3.3 | 1.7 | 2.7 | 0.674 |
| **Preoperative and perioperative conditions** | | |  |  |  |  |
| TC (mmol/L) | 4.17±1.13 | 4.12±1.13 | 4.21±1.12 | 4.18±1.11 | 4.27±1.20 | 0.361 |
| TG (mmol/L) | 1.36[1.01,1.92] | 1.35[1.00,1.89] | 1.32[0.97,1.93] | 1.39[1.05,1.88] | 1.55[1.21,2.14] | 0.074 |
| HDL-C (mmol/L) | 1.02±0.25 | 1.01±0.26 | 1.03±0.24 | 1.01±0.19 | 1.98±0.23 | 0.137 |
| LDL-C (mmol/L) | 2.53±0.95 | 2.49±0.95 | 2.55±0.97 | 2.47±0.84 | 2.66±0.97 | 0.224 |
| CREA (μmol/L) | 72.2±13.7 | 74.9±13.7 | 70.3±13.8 a | 69.7±12.7 b | 70.3±11.5 c | <0.001 |
| eGFR (mL/min/1.73 m^2^) | 92.0±12.4 | 90.6±13.0 | 92.7±12.0 a | 93.8±10.8 b | 94.8±10.2 c | <0.001 |
| eGFR ≥ 90 (mL/min/1.73 m^2^), (%) | 62.9 | 57.7 | 65.1 a | 71.1 b | 73.6 c | <0.001 |
| SUA (μmol/L) | 322.6±82.5 | 362.0±77.1 | 297.9±72.0 a | 274.4±78.8 b,* | 285.0±78.3 c | <0.001 |
| HUA, (%) | 14.1 | 24.1 | 6.9 a | 4.1 b | 8.2 c | <0.001 |
| Na^+^(mmol/L) | 140.399±2.497 | 140.444±2.205 | 140.303±2.673 | 140.741±2.576 | 140.403±2.936 | 0.299 |
| Cl^-^ (mmol/L) | 102.578±2.932 | 102.605±2.798 | 102.545±2.954 | 102.644±2.805 | 102.559±3.735 | 0.973 |
| K^+^ (mmol/L) | 4.116±0.352 | 4.100±0.343 | 4.140±0.354 a | 4.046±0.348 * | 4.124±0.386 | 0.017 |
| Mg^2+^(mmol/L) | 0.904±0.078 | 0.906±0.077 | 0.906±0.079 | 0.897±0.076 | 0.888±0.081 c,† | 0.102 |
| Ca^2+^(mmol/L) | 2.345±0.109 | 2.352±0.112 | 2.342±0.108 | 2.338±0.095 | 2.329±0.104 c | 0.076 |
| hs-CRP(mg/L) | 1.49[0.61,3.85] | 1.46[0.60,3.73] | 1.54[0.57,3.77] | 1.48[0.76,5.11] | 1.40[0.61,4.20] | 0.821 |
| LVEF (%) | 62.0±8.1 | 62.2±7.8 | 62.0±8.1 | 62.2±8.0 | 59.9±9.8 c,† | 0.043 |
| LVEF ≥ 50%, (%) | 93.1 | 93.1 | 94.3 | 94.1 | 83.6 c,†,‡ | 0.001 |
| Preoperative heart rate(bpm) | 75.5±10.3 | 75.4±10.6 | 75.7±10.0 | 75.9±9.6 | 75.0±10.1 | 0.854 |
| Preoperative PR (ms) | 163.2±26.5 | 162.8±26.6 | 163.0±26.1 | 164.9±25.9 | 166.1±28.2 | 0.589 |
| Preoperative QTc (ms) | 432.7±33.8 | 434.1±34.8 | 431.2±33.1 | 433.9±30.9 | 432.7±34.6 | 0.408 |
| Preoperative SBP (mmHg) | 129.5±16.0 | 127.6±14.9 | 129.9±16.5 a | 133.1±16.7 b,* | 134.8±16.5 c,† | <0.001 |
| Preoperative DBP (mmHg) | 75.3±9.8 | 74.6±9.9 | 75.9±9.7 a | 75.1±9.9 | 75.5±9.8 | 0.091 |
| Blood Loss(ml) | 700[600,1000] | 700[600,1000] | 700[600,900] | 800[600,1000] | 800[600,1000] | 0.380 |
| **Postoperative conditions** |  |  |  |  |  |  |
| CREA (μmol/L) | 75.7±16.8 | 74.0±17.5 | 75.8±16.6 a | 78.8±14.4 b | 82.7±12.7 c,† | <0.001 |
| eGFR (mL/min/1.73 m^2^) | 88.0±14.6 | 90.0±14.9 | 87.3±14.5 a | 85.5±12.8 b | 82.7±13.3 c,† | <0.001 |
| SUA (μmol/L) | 246.7±78.8 | 221.5±66.5 | 248.6±72.5 a | 287.7±78.9 b,* | 359.1±80,9 c,†,‡ | <0.001 |
| Na^+^(mmol/L) | 139.879±3.849 | 139.600±3.653 | 139.937±3.775 | 140.591±4.641 b | 140.556±4.495 c | 0.009 |
| Cl^-^ (mmol/L) | 104.603±3.515 | 104.530±3.549 | 104.674±3.352 | 104.776±3.980 | 104.396±3.889 | 0.728 |
| K^+^ (mmol/L) | 4.140±0.373 | 4.137±0.359 | 4.147±0.383 | 4.157±0.375 | 4.094±0.394 | 0.521 |
| Mg^2+^(mmol/L) | 0.834±0.130 | 0.836±0.135 | 0.835±0.129 | 0.819±0.119 | 0.828±0.119 | 0.618 |
| Ca^2+^(mmol/L) | 1.992±0.150 | 1.990±0.154 | 1.993±0.148 | 1.997±0.141 | 1.996±0.153 | 0.946 |
| **In-hospital adverse outcomes** |  |  |  |  |  |  |
| Fatal arrhythmia, (%) | 1.2 | 1.1 | 1.0 | 0.8 | 4.5 c,† | 0.088 |
| All-cause death, (%) | 0.7 | 1.1 | 0.4 | 0.8 | 0.9 | 0.440 |
