# Supplementary figures and images for: The association of perioperative serum uric acid variation with in-hospital adverse outcomes in coronary artery bypass grafting patients
Source: Front Cardiovasc Med. 2024 Oct 1;11:1364744. doi: 10.3389/fcvm.2024.1364744 (PMC11475021; doi:10.3389/fcvm.2024.1364744)

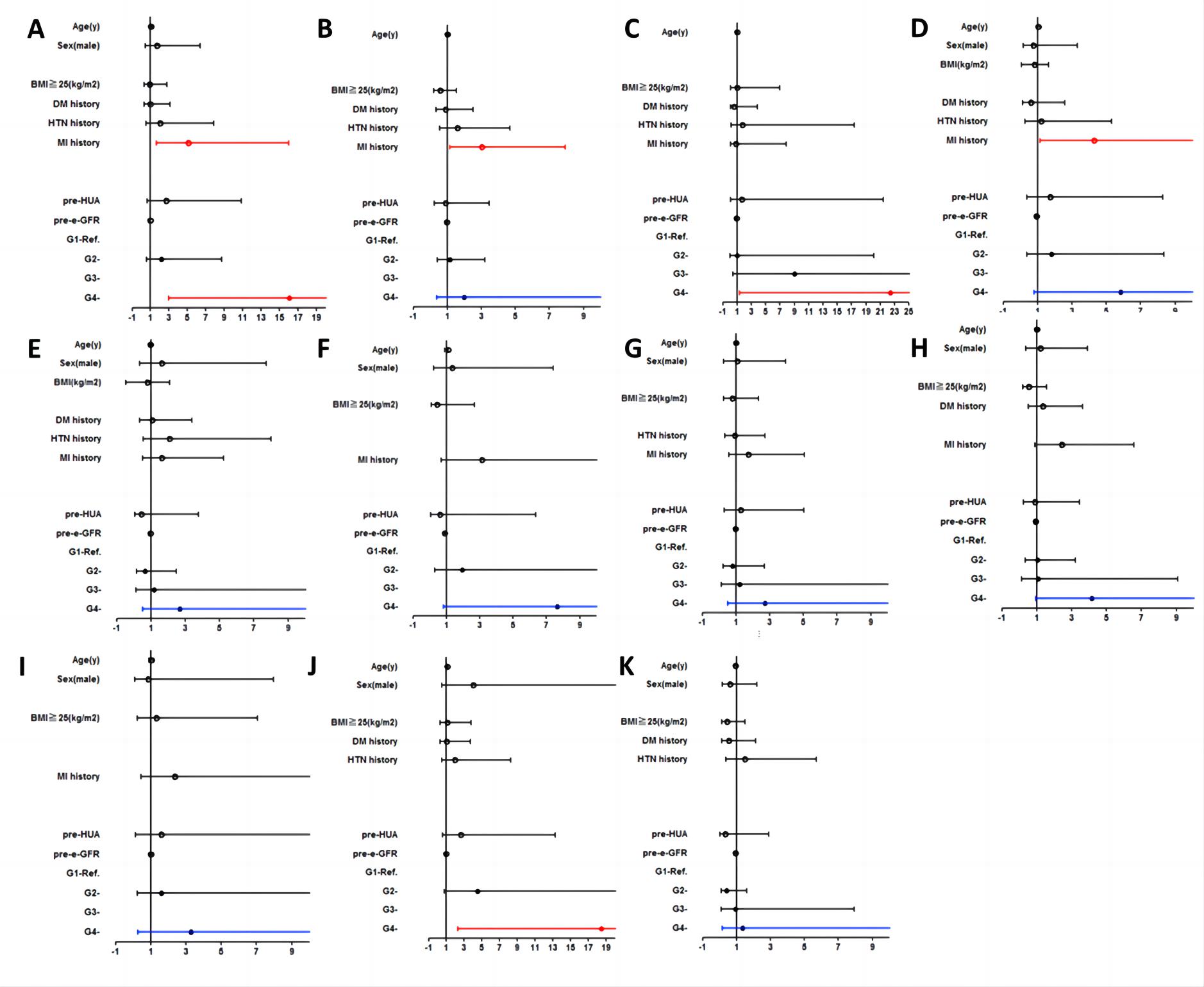

Supplement: Supplementary file 2 [file Image1.jpeg]

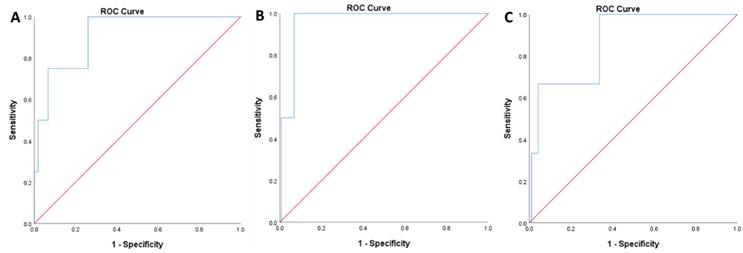

Supplement: Supplementary file 3 [file Image2.jpeg]

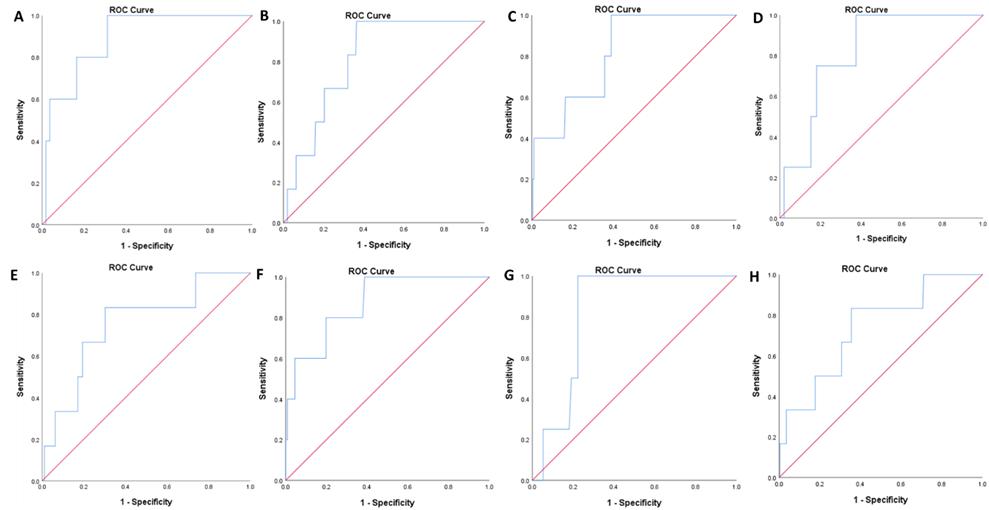

Supplement: Supplementary file 4 [file Image3.jpeg]

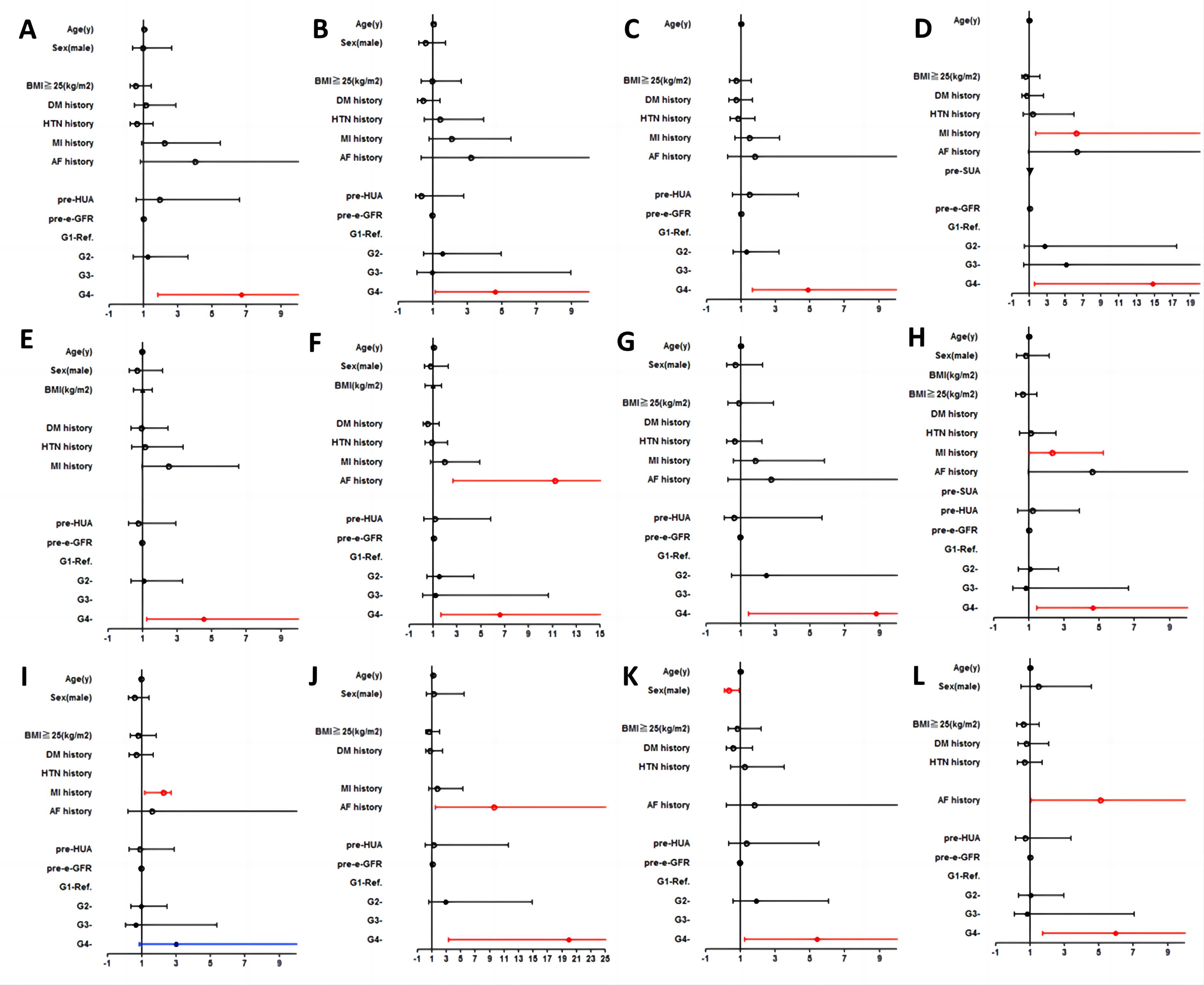

Supplement: Supplementary file 5 [file Image4.jpeg]
